# Prospective Genomic Characterization of the German Enterohemorrhagic *Escherichia coli* O104:H4 Outbreak by Rapid Next Generation Sequencing Technology

**DOI:** 10.1371/journal.pone.0022751

**Published:** 2011-07-20

**Authors:** Alexander Mellmann, Dag Harmsen, Craig A. Cummings, Emily B. Zentz, Shana R. Leopold, Alain Rico, Karola Prior, Rafael Szczepanowski, Yongmei Ji, Wenlan Zhang, Stephen F. McLaughlin, John K. Henkhaus, Benjamin Leopold, Martina Bielaszewska, Rita Prager, Pius M. Brzoska, Richard L. Moore, Simone Guenther, Jonathan M. Rothberg, Helge Karch

**Affiliations:** 1 Institute of Hygiene, University Münster, Münster, Germany; 2 Department of Periodontology, University Münster, Münster, Germany; 3 Life Technologies, Foster City, California, United States of America; 4 OpGen, Gaithersburg, Maryland, United States of America; 5 Life Technologies, Darmstadt, Germany; 6 Robert Koch Institute, Wernigerode Branch, Wernigerode, Germany; 7 Ion Torrent by Life Technologies, Guilford, Connecticut, United States of America; University of Hyderabad, India

## Abstract

An ongoing outbreak of exceptionally virulent Shiga toxin (Stx)-producing *Escherichia coli* O104:H4 centered in Germany, has caused over 830 cases of hemolytic uremic syndrome (HUS) and 46 deaths since May 2011. Serotype O104:H4, which has not been detected in animals, has rarely been associated with HUS in the past. To prospectively elucidate the unique characteristics of this strain in the early stages of this outbreak, we applied whole genome sequencing on the Life Technologies Ion Torrent PGM™ sequencer and Optical Mapping to characterize one outbreak isolate (LB226692) and a historic O104:H4 HUS isolate from 2001 (01-09591). Reference guided draft assemblies of both strains were completed with the newly introduced PGM™ within 62 hours. The HUS-associated strains both carried genes typically found in two types of pathogenic *E. coli*, enteroaggregative *E. coli* (EAEC) and enterohemorrhagic *E. coli* (EHEC). Phylogenetic analyses of 1,144 core *E. coli* genes indicate that the HUS-causing O104:H4 strains and the previously published sequence of the EAEC strain 55989 show a close relationship but are only distantly related to common EHEC serotypes. Though closely related, the outbreak strain differs from the 2001 strain in plasmid content and fimbrial genes. We propose a model in which EAEC 55989 and EHEC O104:H4 strains evolved from a common EHEC O104:H4 progenitor, and suggest that by stepwise gain and loss of chromosomal and plasmid-encoded virulence factors, a highly pathogenic hybrid of EAEC and EHEC emerged as the current outbreak clone. In conclusion, rapid next-generation technologies facilitated prospective whole genome characterization in the early stages of an outbreak.

## Introduction

Enterohemorrhagic *Escherichia coli* (EHEC) are a pathogenic subgroup of Shiga toxin (Stx)-producing *E. coli* (STEC) that cause human disease including diarrhea, bloody diarrhea and the hemolytic uremic syndrome (HUS) [1]. After ingestion of the pathogen and a subsequent incubation period of 2 to 3 days patients (most frequently children) develop watery diarrhea that is typically accompanied by abdominal pain. Bloody diarrhea ensues after a 2–4-day interval in about 80% of cases. Approximately one week (range 3 to 13 days) after the onset of diarrhea, 10% to 15% of patients (data for children under 10 years of age) develop HUS [2], [3]. Since early May 2011, there has been an outbreak of multidrug-resistant EHEC O104:H4 in Germany [4]. As of June 24, 2011, 834 cases of HUS and 2,967 non-HUS cases were reported by the German Robert Koch Institute (RKI); 30 of the HUS cases and 16 of the non-HUS cases resulted in death [5]. Furthermore, 100 additional infections have been identified in 12 other European countries and even in the United States and Canada [6]. While still ongoing at the time of publication, this is already the largest outbreak caused by EHEC in Germany and the largest outbreak of HUS worldwide. Extensive efforts to identify the source implicated contaminated sprouts, which was later confirmed by isolation of the outbreak strain from the sprouts on June 12th [7]. Historically, *E. coli* O104:H4 has been associated with very few HUS cases [8], [9]. To date in Germany only one of 588 EHEC strains isolated from HUS patients in the National Consulting Laboratory for HUS and the Reference Laboratory for *Enterobacteriaceae* of the RKI belongs to serotype O104:H4, and this strain was isolated in 2001 [9]. The *E. coli* O104:H4 isolate from this German HUS case is included in the HUS-associated *E. coli* (HUSEC) collection [9]. This collection presently contains 42 representative, well-characterized EHEC strains that cover the entire serotype and phylogenetic spectrum of HUS-associated EHEC in Germany [9]. Among several different Stx types present in members of the HUSEC collection, Stx2 is most common.

Here, we prospectively use whole genome based methods, including Ion Torrent sequencing [10] and Optical Mapping, during an ongoing outbreak to characterize and compare the *E. coli* O104:H4 outbreak strain with the historical EHEC O104:H4 isolate (01-09591) and EAEC O104:H4 strain 55989, isolated in Central Africa in the late 1990s [11]. These analyses enabled us to propose an evolutionary model for the emergence of the present German outbreak strain, and to identify sequences that are specific to the current outbreak strain.

## Results

### Description of the outbreak

**Figure 1:**
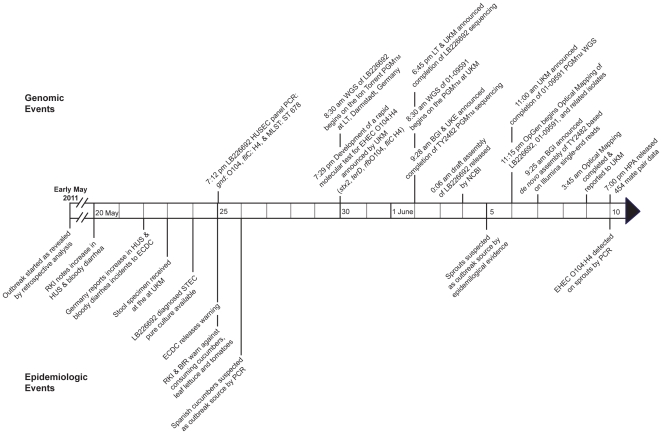
Events timeline of German EHEC O104:H4 outbreak. Major events relating to the outbreak epidemiology (below arrow) and those relating to genomic elucidation efforts (above arrow) are noted separately in the graph. Lines within the arrow indicate single day progression, with the date noted every 5th day. Events span from early May 2011 to early June 2011. Times are noted in Central European Time (CET). Abbreviations: BfR  =  Bundesinstitut für Risikobewertung (Federal Institute for Risk Assessment, Germany), BGI  =  Beijing Genomics Institute (People's Republic of China), ECDC  =  European Center for Disease Prevention and Control (Sweden), HPA  =  Health Protection Agency (United Kingdom), HUS  =  hemolytic uremic syndrome, LT  =  Life Technologies Group, PGM™  =  Ion Torrent Personal Genome Machine™, RKI  =  Robert Koch Institute (Germany), ST  =  multilocus sequence type, UKE  =  University Hospital Hamburg (Germany), UKM  =  University Hospital Muenster (Germany), WGS  =  whole genome sequencing.

Major events in the time course of the outbreak and the genomic elucidation efforts are shown in Figure 1. On May 19, the RKI noted an increased frequency of HUS and bloody diarrhea cases in northern Germany, predominantly among adults [5]. By retrospective analysis this outbreak had begun in early May 2011. The outbreak was reported for the first time to the European Center for Disease Prevention and Control (ECDC) on May 22. On May 25, the RKI and Federal Institute for Risk Assessment (BfR) issued a statement that warned against consuming cucumbers, leaf lettuce, and tomatoes, and the ECDC informed all European countries about the German EHEC outbreak, defined as such based on disease characteristics. One day later, STEC were detected on Spanish cucumbers by PCR, though further investigations ruled out any link to the current O104:H4 outbreak. On June 5, sprouts were suspected as the outbreak source by epidemiological evidence. This was confirmed by detection of EHEC O104:H4 five days later [7]. The efforts to elucidate the source of the outbreak by epidemiological investigations of cases and food supply chains were coordinated by the German Federal Institutions RKI and BfR; microbiological investigations were also coordinated by us as the German National Consulting Laboratory for HUS, a part of the Institute of Hygiene. Although standard methods (stool broth enrichment, subsequent culture on ESBL agar and CT-SMAC, and *stx* PCR) enabled identification of the index isolate, only molecular methods including *gnd* sequencing, MLST, and *fliC* genotyping, enabled us to recognize that the outbreak isolates were very closely related to strain 01-09591 from the HUSEC reference strain collection (The outbreak strain and 01-09591 are collectively referred to here as the ‘HUSEC041 complex’). Subsequently, we used Life Technologies and OpGen next-generation genomic technologies in a prospective manner for the first time ever during an ongoing outbreak [12].

### Relatedness of outbreak and reference isolates

**Figure 2:**
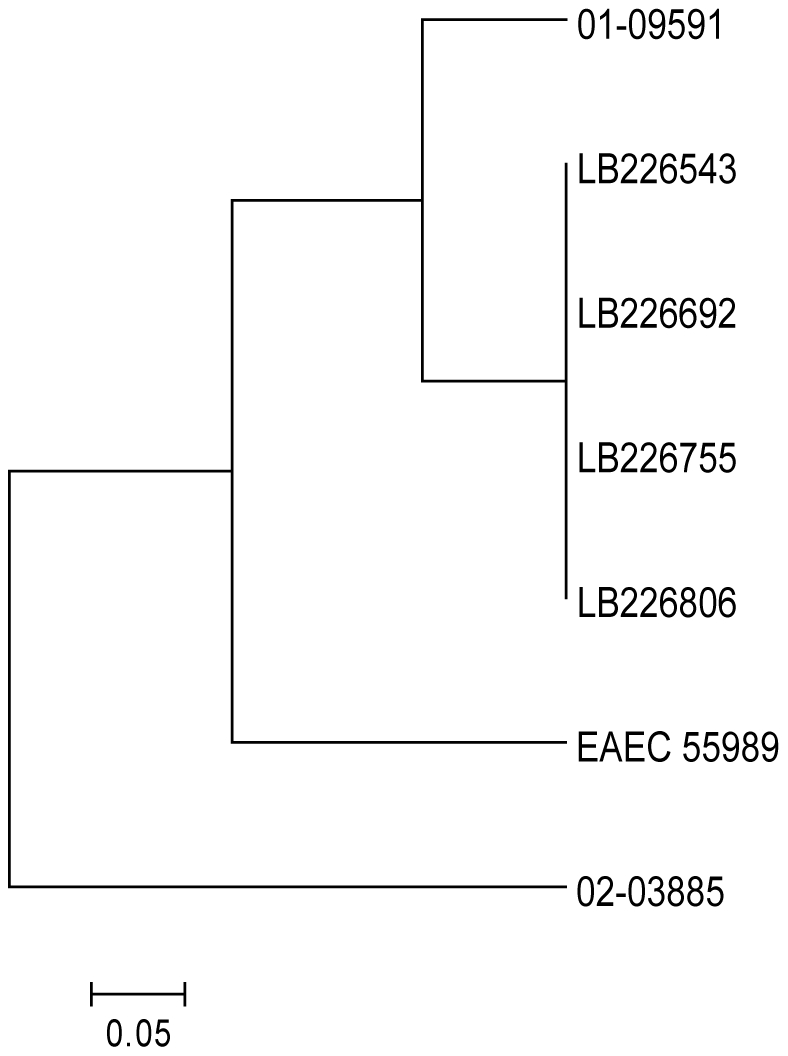
Optical Map similarity cluster of German EHEC O104:H4 outbreak. *De novo* whole genome optical maps from EHEC O104:H4 outbreak strains (‘LB’ prefix) and historical O104:H4 and O104:H21 strains (01-09591 and 02-03885) were created using the Argus™ Optical Mapping System with the *Nco*I restriction enzyme. An *in silico* genomic map of the reference strain 55989 was created in MapSolver™ by importing genomic sequence data from NCBI (acc. no. NC_011478) and applying the *Nco*I restriction pattern. Optical maps and *in silico* maps were compared using the default MapSolver™ parameters and clustered using UPGMA based on the resulting pairwise distance metrics. Scale represents percent difference. Strain name, serotype, country, city, and year of isolation are indicated.

Whole genome optical maps were created for four outbreak isolates (including LB226692) from four different German cities and two historical reference strains 02-03885 (HUSEC037) and 01-09591 (HUSEC041). Comparison of the optical maps demonstrated that all four outbreak strains are identical within the limits of Optical Mapping resolution, suggesting that the outbreak is likely to be clonal and single-sourced (Fig. 2). Moreover, alignments of optical maps to an *in silico* digest of EAEC 55989 showed strong similarity of this strain to the outbreak strains and 01-09591.

### Genomic characterization of the EHEC O104:H4 outbreak strain

**Figure 3:**
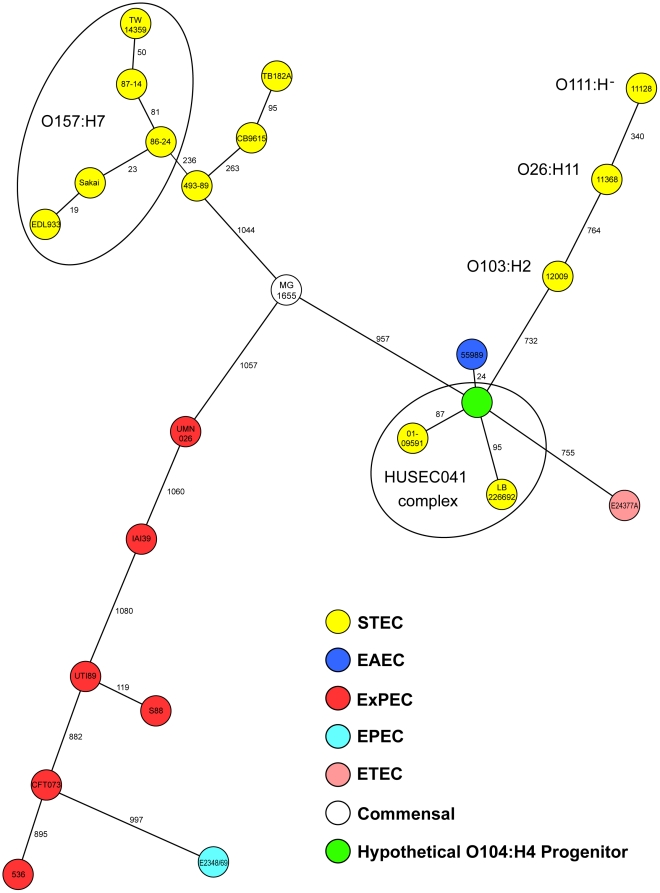
Phylogentic placement of German EHEC O104:H4 outbreak strain. Minimum-spanning tree based on allelic profiles of *E. coli* core genome genes (n = 1,144) portraying the phylogenetic relationship of the EHEC O104:H4 outbreak strain (LB226692), the historical EHEC 01-09591 (HUSEC041), additional *E. coli* strains representing the most common EHEC serotypes, intestinal and extraintestinal *E. coli* pathovars and commensals, from the NCBI RefSeq database. In addition, an *in silico* generated hypothetical O104:H4 progenitor is included. Each dot represents an allelic profile, the number on connecting lines represent the number of alleles that differ between two profiles. The different pathovars (EHEC, EAEC, ExPEC, EPEC, ETEC, commensals) are defined by colors and the EHEC serotypes are indicated.

Sequencing on the Ion Torrent PGM™ sequencer was completed within 62 hours, leading to the public release of the draft assembly of outbreak strain LB226692 on June 3 (Fig. 1, Table S1). Sequence data of the closely related historical isolate 01-09591 was also generated while the outbreak was still occurring. Genome assemblies based on the PGM™ reads showed that both of these HUS-causing strains (LB226692 and 01-09591) carry genes typically found in two different *E. coli* pathotypes, specifically EAEC and EHEC. Genome wide phylogenetic analysis based on core chromosomal ORFs (n = 1,144) demonstrated the close relationship of the LB226692 and 01-09591 strains to the previously sequenced EAEC strain 55989 (NCBI acc. no. NC_011478), and indicated that these strains are only distantly related to the commonly isolated EHEC serotypes (Fig. 3). However, unlike typical EAEC strains, both LB226692 and 01-09591 have an *stx*_2_-harboring prophage integrated in *wrbA*, which is also the integration site for *stx*_2_-phages in EHEC O157:H7 outbreak strains EDL933 [13] and Sakai (RIMD 0509952) [14]. The *wrbA* gene of EAEC 55989 is not occupied by a prophage. Furthermore, the IrgA homologue adhesin encoding gene (*iha*), which is responsible for adherence to epithelial cells and has been found in *eae*-negative STEC [15], is present in all three strains. In contrast to the two HUSEC041 complex strains, 55989 does not harbor the tellurite resistance encoding genes (*ter*). These characteristics led to the development of a rapid PCR-based test of *stx*2, O104 lipopolysaccharide (LPS) gene (*rfb*_O104_), H4 flagellin-encoding gene (*fliC*_H4_), and *terD* for the detection of the HUSEC041 complex [16].

**Figure 4:**
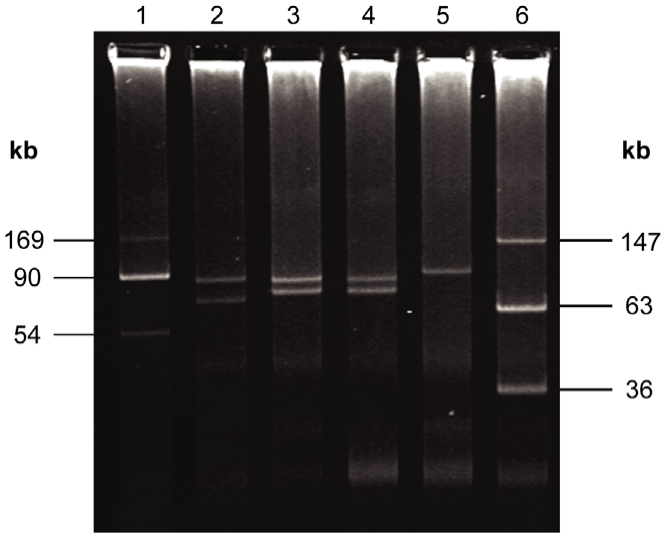
Plasmid profile of German EHEC O104:H4 outbreak strain and strain 01-09591. Comparison of the plasmid content of German EHEC O104:H4 outbreak strains and the 01-09591 (O104:H4; HUSEC041). Lane 1: molecular mass markers (plasmids R27 [169 kb]; R100 [90 kb]; V517 [54 kb]); lane 2: strain 01-09591 EHEC O104:H4; lane 3: German EHEC O104:H4 2011 outbreak strain LB226692; lane 4: German EHEC O104:H4 2011 outbreak strain 11-002097; lane 5: EHEC O157:H7 strain EDL 933; lane 6: E. coli 39R861 molecular size marker.

Plasmid profiling demonstrated that LB226692 and 01-09591 each harbor two large plasmids (Fig. 4; 83 and 90 kb and 75 and 95 kb, respectively). Sequence analysis shows that the smaller plasmid of LB226692 contains aggregative adherence fimbriae type I (AAF/I) but lacks the EAEC heat-stable enterotoxin encoding gene, *astA*. The larger plasmid is an incompatibility group I1 (IncI1) plasmid with high similarity to pEC_Bactec (NCBI acc. no. GU371927) that harbors TEM-1 and CTX-M-15 beta-lactamase genes [17]. The large plasmid of strain 01-09591 appears to be closely related to the IncI1 family plasmid pSERB1 (NCBI acc. no. AY686591) from EAEC strain C1096 [18]. This strain (01-09591) also has a TEM-1 beta-lactamase that is located on a sequence contig carrying a number of genes encoding plasmid functions; we propose that this locus is also carried on the larger plasmid. The smaller plasmid of 01-09591 is an EAEC plasmid containing AAF/III, which is *astA*-positive, and closely related to the plasmid from EAEC 55989. Vitek® 2 and E-test® resistance testing indicates that both strains (LB226692 and 01-09591) have a TEM-1 phenotype. The ESBL genotype (CTX-M-15) of strain LB226692 was also phenotypically confirmed.

In addition to their plasmid content, the current and historical HUS strains differed slightly in chromosomal gene content. Most of the regions of difference were in prophage and insertion elements. One of the prophage regions, present only in 01-09591, harbored a gene encoding a homologue of the EmrE multidrug resistance efflux pump. The strains also differed in their complement of fimbrial operons. LB226692 lacks part of *ybgOPQ*, encoding a putative fimbrial adhesin, and 01-09591 lacks the *fmlA* gene encoding a major fimbrial subunit. Both of these operons are intact in EAEC 55989.

Phenotypic acid resistance testing demonstrated a high number of surviving cells for both strains at pH 2.5 (580 and 540 colony forming units [CFU] per µl liquid culture, LB226692 and 01-09591, respectively), even higher than the number observed for EHEC O157:H7 strain EDL933 (480 CFU/µl). The gene *rpoS*, which is an important regulator for stress response, including acid resistance [19], was found to be identical with an intact reading frame in strains LB226692 and 01-09591.

## Discussion

Rapid next-generation genomics technologies facilitated prospective whole genome characterization in the early stages of this deadly outbreak. Previously, Optical Mapping has been used in retrospective outbreak investigations to examine isolate relatedness and distinguish outbreak from background samples [20]. Development of the rapid, automated Argus™ Optical Mapping System now allows this technique to be used in real time outbreak investigations. In this study, *de novo*, whole-chromosome Optical Maps were created for six organisms in just over two days (Fig. 1).

The Ion Torrent PGM™ sequencing platform [10] makes whole-genome sequencing of microbial isolates in the early stages of an outbreak possible. Using this platform, an outbreak isolate and related strains were sequenced and assembled within two to three days (Fig. 1). A draft assembly of an independent isolate from the same outbreak was simultaneously produced on the PGM™ sequencer over a similar time frame [21]. In addition, by combining whole genome-based next-generation genomics technologies from the laboratory with sophisticated software solutions (e.g., Geographical Information Systems and space-time cluster analysis), highly specific [12] and sensitive real-time monitoring of infectious diseases and early-warning outbreak detection is achievable [22], [23].

The current German outbreak strain is unusual in several aspects. First, Stx-producing serotype O104:H4 are rarely isolated worldwide from HUS patients. In this situation, the availability of the HUSEC collection greatly facilitated the phylogenetic grouping, providing guidance about the virulence profile [9]. Second, there is no evidence of zoonotic origin, in contrast to Stx-producing *E. coli* O104 with H antigens H7, H12 and H21 [24]. Third, the chromosomal backbone is similar to EAEC of serotype O104:H4, which does not cause such severe diseases like HUS. It is also lacking intimin (*eae*), which is responsible for adhesion to epithelial cells in typical EHEC, though it does possess the *iha*, which has been described in other pathogenic *E. coli* lacking intimin [25], [26]. Finally, there have been no previously reported outbreaks caused by this serotype, though another Stx-producing EAEC (Serogroup O111) has been linked to an outbreak of HUS in France [27]. Due to its hybrid pathogenicity characteristics, Brzuszkiewicz and colleagues assigned a new pathotype ‘Entero-Aggregative-Haemorrhagic *Escherichia coli* (EAHEC)’ [3]. While this is an accurate description, we believe that grouping of the current outbreak strains into the pathotype ‘EHEC’ is appropriate as it reflects the major clinical attributes and the follows the precedent set by other *eae*-negative EHEC (e. g. O91:H21, O113:H21, O121:H19) [28].

**Figure 5:**
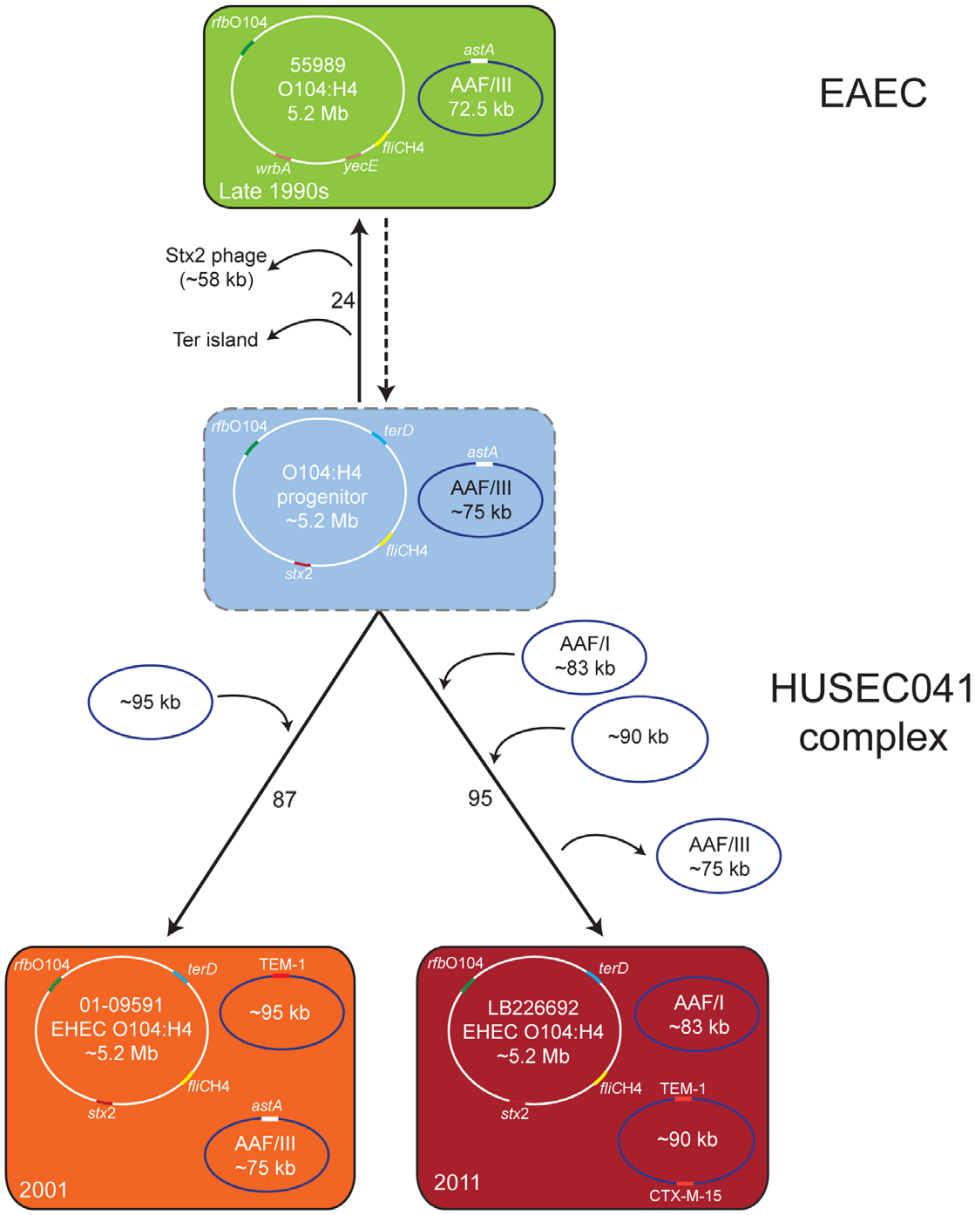
Evolutionary model of the origin of the German EHEC O104:H4 outbreak strain. Evolutionary model of the current outbreak strain (LB226692) and the historical strain (01-09591) from 2001 based on whole chromosomal and plasmid data. Numbers on connecting lines indicate the number of loci that differ between the strains as determined by analysis of 1,144 core genome genes. The genes of the PCR test for differentiation of the HUSEC041 complex (*stx*2: red; *terD*: blue; *rfb*_O104_: green; *fliC*_H4_: yellow), [16] for antibiotic resistance (orange: TEM-1 and CTX-M-15), and for the differentiation of EAEC plasmids [5] (*astA*: white) are colored. The order of plasmid acquisition and loss were arbitrarily chosen in the illustration as the exact sequence of events is not known. Year of isolation for each strain is noted in the lower left corner of each rectangle.

An evolutionary model of the origin of the present outbreak strain (Fig. 5) proposes that a hypothetical Stx-producing *E. coli* O104:H4 with an EAEC genetic background gave rise to both HUSEC041 strains (LB226692 and 01-09591) and the EAEC strain 55989. In principle, there are two evolutionary models possible: (i) the ‘common ancestor model’ proposes a hypothetical O104:H4 progenitor and (ii) the ‘linear ancestry model’ suggests that all EHEC O104:H4 originated from the prototypical EAEC 55989. Inclusion of the sequence information of the historical EHEC O104:H4 strain (01-09591) provides additional information supporting the common ancestor model. Acquisition of Stx-encoding genes in strains with an unoccupied insertion site has been previously shown [29], however, loss of several genes and genomic islands is more likely and occurs frequently [30], [31]. Therefore, EAEC 55989 (*E. coli* O104:H4, ST678) appears to be recently derived from a progenitor Stx-producing *E. coli* O104:H4. Only 24 out of a total of 1,144 core genes vary in primary sequence between these strains (Fig. 5). Moreover, the existence of an intact *stx* integration site at *wrbA* in EAEC 55989 and the presence of *iha*, which is often adjacent to tellurite resistance genes [31], further corroborates the model of descent from the putative HUSEC041 progenitor. The HUSEC041 complex is *terD* positive, but we recently showed that the *ter* island can frequently be lost via complete or internal deletions in *ter-*harboring O island [31]. Such profound chromosomal changes can occur during the brief period that EHEC passes through the human gastrointestinal tract leading to gains and losses of virulence determinants, which may account for the multiple loci differences and plasmid gain and loss seen in this model [1]. In almost all major EHEC serotypes *stx*2 loss has been described, leading to enormous difficulties in the diagnosis of these pathogens [30]. Both isolates of the HUSEC041 complex had 87 (01-09591) and 95 (LB226692) unique core genome alleles which possibly can be attributed to the additional time to acquire mutations since divergence from the common progenitor as these were isolated in 2001 and 2011, respectively (Fig. 5). To visualize the common ancestor model in the phylogenetic tree, we have incorporated the hypothetical O104:H4 progenitor in figure 3.

**Figure 6:**
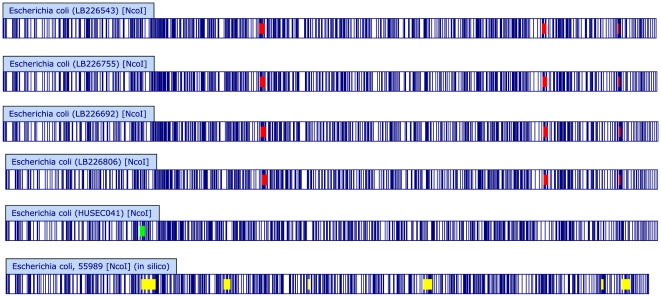
Whole chromosomal Optical Maps of the EHEC O104:H4 outbreak and related strains. Optical Maps were created from current outbreak isolates (LB isolates) and 01-09591 using *Nco*I as described. An *in silico* reference map was also created from the published EAEC 55989 sequence [11]. Optical and *in silico* maps were compared to reveal shared and unique elements. Shared restriction fragments are white/un-highlighted. Regions shared by the current outbreak isolates but unique relative to 01-09591 and EAEC 55989 are highlighted in red, regions unique to 01-09591 are highlighted in green, and regions unique to EAEC 55989 are highlighted in yellow. Perceived minor variations in banding patterns are due to fragment sizes less than 2 kb and therefore not included in subsequent analysis (see Methods).

Optical Mapping data using four outbreak strains, 01-09591 and EAEC 55989 also corroborates the common ancestor model (Fig 6). If it is assumed that the hypothetical common O104:H4 ancestor contains the shared genomic regions of the outbreak strains, then LB226692 evolved by three insertion events, 01-09591 by one insertion event, and EAEC 55989 by six insertion events. All of these events would be unconstrained, meaning that the nature and location of the insertions leading to one strain would be independent of the insertions that lead to the other strains. According to the linear model, following the time of isolation, EAEC 55989 leads to 01-09591, which then leads to LB226692. In this model, EAEC 55989 loses six regions and gains one new genomic region to give rise to 01-09591. Then, 01-09591 loses this recently gained region and gains three additional new regions leading to the current outbreak strain. Although the total number of genomic changes is the same in both models, the linear model imposes an additional constraint of the gain and subsequent loss of the same genetic region.

Interestingly, the isolate from 2001 retained the 75 kb plasmid from the progenitor encoding AAF/III fimbriae (also present in EAEC strain 55989 in a different size) and acquired a 95 kb plasmid encoding type IV pilus system and TEM-1. In contrast, the outbreak isolate LB226692 acquired two new plasmids, one encoding AAF/I fimbriae (83 kb) and another encoding TEM-1 and CTX-M-15 beta-lactamases (90 kb), and lost the AAF/III fimbriae encoding plasmid. Whereas the ESBL phenotype is an additional characteristic of the outbreak strain, the remaining antibiotic susceptibility profile is similar to the isolate from 2001 (http://www.ehec.org). Finally, the high acid resistance of the HUSEC041 complex strains may facilitate survival during passage through the acidic environment of the stomach, thus contributing to the high pathogenicity of the current outbreak strain.

In conclusion, rapid next-generation technologies facilitated prospective whole genome characterization in the early stages of an outbreak. In the future, these technologies will help to make informed decisions about treatment, prevention, and source tracking.

## Materials and Methods

### Patients and strains

The outbreak isolate LB226692 and the historic isolates 01-09591 (HUSEC041; serotype O104:H4) were selected for whole genome sequencing. LB226692 originates from a HUS patient from Paderborn (Germany), who was associated with the current outbreak. The stool from this patient was received by the German National Consulting Laboratory for HUS at the Institute of Hygiene, University of Münster, on May 23. LB226692 was isolated on May 24, 2011, using stool enrichment in GN broth (Hajna) followed by plating the enriched cultures on extended spectrum beta-lactamase (ESBL) agar (chromID ESBL, bioMérieux, Nürtingen, Germany) and cefixime-tellurite sorbitol MacConkey agar (CT-SMAC, Oxoid, Wesel, Germany). Analyses of the isolate using PCR on May 24, 2011 detected a *stx*_2_-positive (99% homologous to stx2 sequence of prototypic Shiga toxin- producing *E. coli* O157:H7 strain EDL933 with one synonymous nucleotide change in each subunit gene) [16], *eae*-negative EHEC, that was one day later subtyped as *gnd* O104, *fli*C H4 and multilocus sequence typing (MLST) sequence type (ST) 678 [32]-[34]. The strain 01-09591 (*gnd* O104, *fliC* H4, ST678) was isolated from an HUS patient in Germany in 2001 and is part of the HUSEC collection [9].

### DNA preparation, sequencing, assembly, and phylogenetic analysis

Genomic DNA of strains LB226692 and 01-09591 (HUSEC041) was isolated from 1.5 ml of liquid cultures (37°C overnight) following published protocol [35] with slight modifications; i.e., lysis time increased to 1.5 hours and no phenol/chloroform/isoamylalcohol precipitation step. Concentration of reconstituted genomic DNA was determined with the Qubit dsDNA BR Assay (Life Technologies, Invitrogen division, Darmstadt, Germany). Library preparation was performed with the Ion Fragment Library Kit (Life Technologies, Darmstadt, Germany) according to the protocol (part no. 4467320 rev. A, 04/2011) with minor modifications. Size selection was done with E-Gel® Size Select 2% Agarose (Invitrogen) for strain LB226692 or with Caliper LabChip® XT (Caliper Life Sciences, Mainz, Germany) for strain 01-09591. All quality control analyses for the 01-09591 library were performed with a Caliper LabChip® GX using the DNA High Sensitivity Assay (Caliper Life Sciences). Template preparation was carried out with the Ion Xpress™ Template Kit (Life Technologies) according to the Ion Xpress™ Template Kit User Guide (part no. 4467389 Rev. B, 05/2011) with a modified protocol for Ion Sphere™ recovery (Recovery Steps 1.f to 4.b). Emulsified Ion Sphere™ particles were collected by centrifugation (2200 g for 8 min) in a SOLiD® emulsion collection tray (Life Technologies). After centrifugation a clear oil phase developed above a white solid pellet. The oil layer was decanted and pelleted Ion Spheres were resuspended with 700 µl of breaking solution followed by two washes of the emulsion collection tray with breaking solution. In a departure from the User Guide, all three fractions were pooled in the same 2.0 ml reaction tube. Washing of the recovered Ion Sphere particles was performed as described in the original protocol (Steps 4.c and following). The Ion Sequencing Kit (Life Technologies) was used with the Personal Genome Machine™ (PGM™) sequencer as described in the Ion Sequencing Kit User Guide (part no. 4467391 rev. B, 04/2011). Enriched ISPs were prepared for sequencing as described in the protocol and deposited on the chip in three consecutive loading cycles. Each cycle was composed of the following steps: (i) adjust sample volume to 19 µl with annealing buffer, (ii) 10 sec sonication followed by a quick spin, (iii) re-suspension by pipetting and loading of 6 µl of the sample to the chip, and (iv) 3 min centrifugation using the custom centrifuge adapter/rotor. In total ten 314-chip sequencing runs (65 cycles per run) were performed with the LB226692 library as a template and seven 314-chip sequencing runs with the 01-09591 library.

The Ion Torrent reads were assembled using the publicly available EAEC 55989 genome sequence [11] in a reference-guided strategy. PGM™ sequencer reads were aligned to the 55989 chromosome (GenBank acc. NC_011478) and plasmid (NC_011752) with TMAP [36]. A consensus sequence was generated with SAMtools [37] and split at zero-coverage gaps using a custom Perl script to generate consensus contigs. Subsequently, MIRA (v. 3.2.17_dev) was used for a “backbone” assembly in which reads were first mapped to the consensus contigs and then the unmapped reads were used in a *de novo* assembly to fill gaps and unique regions not present in the reference genome [38]. Finally, some of the contigs were merged with CAP3 [39]. The draft genome assemblies are deposited at NCBI as AFOB00000000 (LB226692) and AFPS00000000 (01-09591). Additionally, MIRA was used in the absence of a reference sequence to generate alternate contigs based purely on de novo assembly methods.

Phylogenetic analysis of the *E. coli* core genome was performed on the basis of published *E. coli* sequences from NCBI RefSeq representing the different *E. coli* pathovars and commensals. Using Perl scripts, all ORFs from these genomes were extracted on the nucleotide level and BLASTed against each other with thresholds of 95% nucleotide similarity and 100% overlap; duplicates were excluded. The core genome was then defined as the ORFs that were present in all *E. coli* analyzed (see Table S2). The resulting 1,144 core genome ORFs were subsequently imported into a locally installed and modified version of the BIGSdb software [40]. Finally, the published genome sequences used for core genome definition and the newly determined sequences were uploaded into the BIGSdb database and allelic designations were given for each orthologous sequence of each ORF using default parameters except “minimum similarity” and “overlap” were set to 95%. To compensate for homopolymer sequencing errors, all sequences were controlled for indels by comparison with the core genome genes and homopolymers were manually corrected by majority rule in 51 of the 1,144 genes. Allelic profiles were exported from the database and the minimum spanning tree based on allelic profiles was constructed using RIDOM MST™ version 0.9 beta (Ridom GmbH, Münster, Germany).

### Optical Mapping and plasmid profiles

Four outbreak isolates (LB226692, LB226755, LB226806, and LB226543) from the first four German cities that sent samples to the National Consulting Laboratory for HUS (Paderborn, Frankfurt, Hamburg, and Münster, respectively) and two historical reference strains 02-03885 (HUSEC037, serotype O104:H21, ST672) and 01-09591 (HUSEC041) were chosen for optical mapping. Chromosomal DNA was digested using *Nco*I and optical map production was carried out with the Argus™ Optical Mapping System (OpGen Inc., Gaithersburg, MD). Optical Map comparisons were performed similarly to the method described by Schwan et al. [41]. Briefly, each pair of Optical Maps was optimally aligned using the dynamic programming algorithm implemented in MapSolver™ software (OpGen). The score for each alignment was proportional to the log of the length of the alignment minus a penalty that incorporates fragment sizing errors, false cuts, missing cuts, and loss of small fragments; hence, longer alignments between more similar patterns produced higher scores. MapSolver™ was also used to generate similarity clusters. In summary, each map was first aligned to every other map. From these alignments, a pairwise percent dissimilarity score was calculated and these scores were used as inputs into an agglomerative clustering method using UPGMA. These dissimilarity measurements were used as inputs into an agglomerative clustering method using UPGMA. Large plasmids were isolated and visualized by agar electrophoresis as published in literature [42].

### Antibiotic and acid resistance testing

Antibiotic resistance for strains LB226692 and 01-09591 was determined using the Vitek® 2 (bioMérieux), system version 05.02. MICs were interpreted in accordance with the EUCAST 2010 guidelines. Extended-spectrum beta-lactamase (ESBL) production was confirmed using E-tests® for piperacillin, piperacillin/tazobactam, cefotaxime, cefotaxime/clavulanic acid, ceftazidime, and ceftazidime/clavulanic acid (bioMérieux). Acid resistance at pH 2.5 for strains LB226692 and 01-09591 was tested as previously published [43] with an inoculum of 20,000 CFU/µl and EHEC O157:H7 strain EDL933 [19] as a control.

## Supporting Information

Table S1 **Summary of Ion Torrent PGM™ sequencing and assembly metrics.**
(DOC)

Table S2 **List of core genome genes (n = 1,144) used for phylogenetic analysis.**
(DOC)
