# Supplementary material for: Prospective Genomic Characterization of the German Enterohemorrhagic Escherichia coli O104:H4 Outbreak by Rapid Next Generation Sequencing Technology
Source: PLoS One. 2011 Jul 20;6(7):e22751. doi: 10.1371/journal.pone.0022751 (PMC3140518; doi:10.1371/journal.pone.0022751)
Supplement: Table S1 — Summary of Ion Torrent PGM™ sequencing and assembly metrics. (DOC) [file pone.0022751.s001.doc]

**Supporting Information Table S1. Summary of Ion Torrent PGM^TM^ sequencing and assembly metrics.**

| **Strains**  **Sequencing and assembly metrics** | **LB226692** | **01-09591** |
| --- | --- | --- |
| 314 Ion Torrent PGM^TM^ chip runs | 10 | 7 |
| Coverage | 21.07 | 22.04 |
| A% | 24.7 | 24.68 |
| C% | 25.35 | 25.37 |
| G% | 25.26 | 25.22 |
| T% | 24.66 | 24.66 |
| N% | 0.02 | 0.07 |
| Sum contig length | 5,482,130 | 5,516,113 |
| Num contigs | 555 | 456 |
| Mean contig length | 9,877 | 12,096 |
| Median contig length | 317 | 268 |
| N50 contig length | 175,824 | 267,769 |
| N90 contig length | 16,305 | 23,521 |
| N95 contig length | 4,262 | 6,102 |
| Max contig length | 475,711 | 868,354 |
