# Supplementary material for: Prospective Genomic Characterization of the German Enterohemorrhagic Escherichia coli O104:H4 Outbreak by Rapid Next Generation Sequencing Technology
Source: PLoS One. 2011 Jul 20;6(7):e22751. doi: 10.1371/journal.pone.0022751 (PMC3140518; doi:10.1371/journal.pone.0022751)
Supplement: Table S2 — List of core genome genes (n = 1,144) used for phylogenetic analysis. (DOC) [file pone.0022751.s002.doc]

**Supporting Information Table S2. *E. coli* core genes used in this study.** Gene nomenclature is based upon strain O157 Sakai. Locus tag, start and stop coordinates and gene product are listed.

| Locus tag | Start coordinate | Stop coordinate | Gene product |
| --- | --- | --- | --- |
| ECs0002 | 354 | 2813 | aspartokinase I-homoserine dehydrogenase I |
| ECs0003 | 2818 | 3747 | homoserine kinase |
| ECs0004 | 3751 | 5034 | threonine synthase |
| ECs0006 | 5703 | 6476 | hypothetical protein |
| ECs0007 | 6549 | 7976 | putative inner membrane transport protein |
| ECs0008 | 8255 | 9205 | transaldolase B |
| ECs0010 | 9948 | 10511 | hypothetical protein |
| ECs0013 | 11402 | 11803 | hypothetical protein |
| ECs0014 | 12180 | 14093 | heat shock protein DnaK |
| ECs0026 | 25223 | 25483 | 30S ribosomal subunit protein S20 |
| ECs0028 | 25818 | 26756 | putative regulator |
| ECs0030 | 29618 | 30109 | prolipoprotein signal peptidase |
| ECs0031 | 30200 | 30646 | putative FKBX-type 16KD peptidyl-prolyl cis-trans isomerase |
| ECs0037 | 38673 | 39065 | transcriptional regulator of cai operon |
| ECs0041 | 42307 | 43521 | l-carnitine dehydratase |
| ECs0042 | 43653 | 44792 | putative carnitine operon oxidoreductase |
| ECs0046 | 48593 | 49876 | flavoprotein |
| ECs0047 | 49876 | 50160 | putative ferredoxin |
| ECs0048 | 50221 | 51549 | putative transport protein |
| ECs0049 | 51660 | 52187 | putative NAD(P)H oxidoreductase |
| ECs0055 | 56207 | 56581 | hypothetical protein |
| ECs0056 | 56587 | 57405 | dimethyladenosine transferase |
| ECs0062 | 64622 | 65278 | hypothetical protein |
| ECs0063 | 65293 | 68196 | probable ATP-dependent RNA helicase |
| ECs0066 | 71684 | 73183 | L-arabinose isomerase |
| ECs0067 | 73197 | 74894 | L-ribulokinase |
| ECs0069 | 76197 | 76958 | hypothetical protein |
| ECs0076 | 84074 | 85471 | 3-isopropylmalate isomerase (dehydratase) subunit |
| ECs0084 | 92633 | 93634 | transcriptional repressor of fru operon and others |
| ECs0086 | 94699 | 95637 | putative apolipoprotein |
| ECs0087 | 95637 | 95999 | cell division protein FtsL |
| ECs0088 | 96018 | 97781 | septum formation protein FtsI |
| ECs0089 | 97771 | 99255 | meso-diaminopimelate-adding enzyme |
| ECs0092 | 101692 | 103005 | UDP-N-acetylmuramoylalanine-D-glutamate ligase |
| ECs0094 | 104249 | 105313 | UDP-N-acetylglucosamine:N-acetylmuramyl- (pentapeptide) pyrophosphoryl-undecaprenol N-acetylglucosamine transferase |
| ECs0095 | 105370 | 106842 | UDP-N-acetyl-muramate:alanine ligase |
| ECs0097 | 107760 | 108587 | cell division protein FtsQ |
| ECs0098 | 108587 | 109846 | ATP-binding cell division protein FtsA |
| ECs0099 | 109910 | 111058 | cell division protein FtsZ |
| ECs0100 | 111162 | 112076 | UDP-3-O-acyl N-acetylglucosamine deacetylase |
| ECs0106 | 116348 | 117088 | hypothetical protein |
| ECs0107 | 117091 | 117708 | putative DNA repair protein |
| ECs0108 | 117933 | 118973 | GMP reductase |
| ECs0112 | 121601 | 122038 | prelipin peptidase dependent protein |
| ECs0114 | 123222 | 123770 | AmpD protein |
| ECs0117 | 126581 | 127342 | transcriptional regulator for pyruvate dehydrogenase complex |
| ECs0122 | 135987 | 138581 | aconitate hydrase B |
| ECs0124 | 139163 | 139954 | S-adenosylmethionine decarboxylase |
| ECs0125 | 139973 | 140836 | spermidine synthase |
| ECs0130 | 146383 | 147042 | putative carbonic anhdrase |
| ECs0131 | 147151 | 148074 | putative ATP-binding component of a transport system |
| ECs0135 | 150689 | 151066 | aspartate 1-decarboxylase |
| ECs0149 | 164474 | 164926 | dnaK suppressor protein |
| ECs0150 | 165107 | 165808 | probable regulator for maltose metabolism |
| ECs0153 | 169052 | 171583 | peptidoglycan synthetase MrcB |
| ECs0155 | 174100 | 174894 | ATP-binding component of hydroxymate-dependent iron transport |
| ECs0161 | 181203 | 181823 | hypothetical protein |
| ECs0163 | 182657 | 183352 | hypothetical protein |
| ECs0164 | 183436 | 184950 | deoxyguanosine triphosphate triphosphohydrolase |
| ECs0165 | 185083 | 186504 | periplasmic serine protease Do; heat shock protein HtrA |
| ECs0168 | 188612 | 189433 | 2,3,4,5-tetrahydropyridine-2-carboxylate N-succinyltransferase |
| ECs0170 | 192201 | 192992 | methionine aminopeptidase |
| ECs0171 | 193360 | 194082 | 30S ribosomal subunit protein S2 |
| ECs0172 | 194343 | 195191 | protein chain elongation factor EF-Ts |
| ECs0173 | 195341 | 196063 | uridylate kinase |
| ECs0174 | 196216 | 196770 | ribosome releasing factor |
| ECs0179 | 201272 | 203701 | hypothetical protein |
| ECs0180 | 203825 | 204307 | histone-like protein HlpA |
| ECs0181 | 204314 | 205336 | UDP-3-O-(3-hydroxymyristoyl)-glucosamine N-acyltransferase |
| ECs0183 | 205903 | 206688 | UDP-N-acetylglucosamine acetyltransferase |
| ECs0197 | 222220 | 222924 | hypothetical protein |
| ECs0198 | 222924 | 223325 | regulator in colanic acid synthesis RcsF |
| ECs0200 | 224301 | 224951 | putative transport system permease protein |
| ECs0201 | 224947 | 225975 | ATP-binding component of a transporter |
| ECs0207 | 236150 | 237505 | transcriptional regulator for nitrite reductase (cytochrome c552) |
| ECs0208 | 237580 | 238332 | probable hydroxyacylglutathione hydrolase |
| ECs0249 | 281611 | 282186 | phosphoheptose isomerase |
| ECs0251 | 283035 | 283772 | hypothetical protein |
| ECs0264 | 292149 | 293603 | aminoacyl-histidine dipeptidase PepD |
| ECs0265 | 293864 | 294319 | guanine-hypoxanthine phosphoribosyltransferase |
| ECs0266 | 294414 | 295655 | hypothetical protein |
| ECs0269 | 297499 | 298599 | gamma-glutamate kinase |
| ECs0344 | 364322 | 365038 | putative dehydrogenase subunit |
| ECs0345 | 365052 | 366476 | hypothetical protein |
| ECs0360 | 377127 | 379157 | high-affinity choline transport |
| ECs0383 | 403183 | 403455 | hypothetical protein |
| ECs0386 | 406427 | 407593 | putative citrate synthase |
| ECs0387 | 407630 | 409078 | hypothetical protein |
| ECs0429 | 458807 | 459112 | hypothetical protein |
| ECs0431 | 459611 | 460702 | D-alanine-D-alanine ligase A |
| ECs0432 | 461165 | 461422 | hypothetical protein |
| ECs0435 | 463482 | 464594 | hypothetical protein |
| ECs0437 | 464617 | 465423 | pyrroline-5-carboxylate reductase |
| ECs0438 | 466184 | 466705 | shikimate kinase II |
| ECs0439 | 466758 | 466946 | hypothetical protein |
| ECs0440 | 467207 | 467881 | aroM protein |
| ECs0441 | 467956 | 468237 | hypothetical protein |
| ECs0444 | 471197 | 472105 | putative NAGC-like transcriptional regulator |
| ECs0445 | 472230 | 473135 | recombination associated protein |
| ECs0450 | 479870 | 481162 | Positive and Negative sensor protein for pho regulon PhoR |
| ECs0451 | 481572 | 482888 | branched chain amino acid transport system II carrier protein |
| ECs0456 | 488641 | 489708 | S-adenosylmethionine--tRNA ribosyltransferase-isomerase |
| ECs0457 | 489766 | 490890 | tRNA-guanine transglycosylase |
| ECs0458 | 490916 | 491245 | hypothetical protein |
| ECs0466 | 497257 | 497703 | hypothetical protein |
| ECs0468 | 498902 | 499369 | riboflavin synthase beta chain |
| ECs0470 | 499889 | 500863 | thiamin-monophosphate kinase |
| ECs0474 | 504371 | 506230 | 1-deoxy-D-xylulose 5-phosphate synthase |
| ECs0476 | 507157 | 507396 | exonuclease VII small subunit |
| ECs0479 | 509660 | 510568 | involved in thiamin biosynthesis, alternative pyrimidine biosynthesis |
| ECs0484 | 514102 | 514713 | cytochrome o ubiquinol oxidase subunit III |
| ECs0485 | 514706 | 516694 | cytochrome o ubiquinol oxidase subunit I |
| ECs0487 | 518126 | 519598 | regulates beta-lactamase synthesis |
| ECs0489 | 520525 | 520839 | putative regulator of murein genes |
| ECs0491 | 522729 | 523349 | ATP-dependent proteolytic subunit of clpA-clpP serine protease ClpP |
| ECs0492 | 523478 | 524749 | ATP-dependent specificity component of clpP serine protease ClpX |
| ECs0494 | 527503 | 527772 | DNA-binding protein HU-1 |
| ECs0495 | 527967 | 529835 | putative protease maturation protein |
| ECs0496 | 529989 | 530357 | hypothetical protein |
| ECs0497 | 530466 | 530861 | hypothetical protein |
| ECs0498 | 530919 | 531611 | hypothetical protein |
| ECs0504 | 538661 | 538996 | nitrogen regulatory protein P-II 2 |
| ECs0505 | 539029 | 540312 | probable ammonium transporter |
| ECs0509 | 542737 | 543087 | hypothetical protein |
| ECs0514 | 546402 | 546773 | hypothetical protein |
| ECs0523 | 557987 | 559915 | DNA polymerase III tau and gamma subunits |
| ECs0525 | 560300 | 560902 | recombination protein RecR |
| ECs0526 | 561015 | 562886 | chaperone Hsp90 HtpG |
| ECs0528 | 563846 | 564805 | ferrochelatase |
| ECs0530 | 565916 | 567217 | inosine-guanosine kinase |
| ECs0577 | 635772 | 636554 | hypothetical protein |
| ECs0583 | 642947 | 643837 | putative carbamate kinase |
| ECs0584 | 643982 | 645046 | phosphoribosylaminoimidazole carboxylase |
| ECs0587 | 646398 | 646889 | peptidyl-prolyl cis-trans isomerase B |
| ECs0590 | 649116 | 649325 | hypothetical protein |
| ECs0591 | 649330 | 650193 | 5,10-methylene-tetrahydrofolate dehydrogenase |
| ECs0611 | 679666 | 679995 | hypothetical protein |
| ECs0612 | 680014 | 681234 | putative resistance protein |
| ECs0616 | 687290 | 687940 | oxygen-insensitive NAD(P)H nitroreductase |
| ECs0618 | 688470 | 688715 | hypothetical protein |
| ECs0619 | 688784 | 689899 | hypothetical protein |
| ECs0627 | 701043 | 701855 | ATP-binding component of ferric enterobactin transport |
| ECs0634 | 709164 | 710018 | 2,3-dihydro-2,3-dihydroxybenzoate synthetase |
| ECs0636 | 710770 | 711180 | hypothetical protein |
| ECs0644 | 720415 | 720975 | alkyl hydroperoxide reductase C22 subunit |
| ECs0649 | 724914 | 725321 | regulator of nucleoside diphosphate kinase |
| ECs0653 | 728841 | 729389 | hypothetical protein |
| ECs0654 | 729396 | 730925 | citrate lyase alpha chain |
| ECs0660 | 735941 | 737323 | c4-dicarboxylate anaerobic carrier DcuC |
| ECs0663 | 738913 | 739293 | hypothetical protein |
| ECs0665 | 740303 | 740503 | Sec-independent protein translocase |
| ECs0666 | 740610 | 741572 | lipoate synthesis protein LipA |
| ECs0668 | 742997 | 743635 | lipoate biosynthesis protein LipB |
| ECs0669 | 743739 | 743999 | hypothetical protein |
| ECs0671 | 745463 | 746548 | a minor lipoprotein |
| ECs0673 | 747677 | 749575 | peptidoglycan synthetase penicillin-binding protein 2 |
| ECs0674 | 749609 | 750073 | hypothetical protein |
| ECs0675 | 750080 | 750394 | hypothetical protein |
| ECs0678 | 751935 | 752963 | DNA polymerase III delta subunit |
| ECs0681 | 756377 | 756856 | putative alpha helical protein |
| ECs0690 | 764840 | 765772 | putative tRNA synthetase |
| ECs0692 | 766618 | 767289 | glutamate/aspartate transport system permease |
| ECs0693 | 767292 | 768029 | glutamate/aspartate transport system permease |
| ECs0696 | 771070 | 771945 | putative transport protein |
| ECs0697 | 772038 | 772502 | hypothetical protein |
| ECs0699 | 773695 | 775116 | hypothetical protein |
| ECs0705 | 779600 | 780349 | N-acetylglucosamine metabolism |
| ECs0706 | 780400 | 781617 | transcriptional repressor of nag (N-acetylglucosamine) operon |
| ECs0708 | 782837 | 783634 | glucosamine-6-phosphate deaminase |
| ECs0710 | 786116 | 787777 | glutamine tRNA synthetase |
| ECs0714 | 790227 | 790670 | Negative regulator |
| ECs0717 | 792065 | 792826 | hypothetical protein |
| ECs0718 | 793011 | 793553 | Negative modulator of initiation of replication |
| ECs0725 | 803418 | 805463 | high-affinity potassium-transporting ATPase B chain |
| ECs0728 | 807561 | 807764 | hypothetical protein |
| ECs0735 | 819473 | 820213 | hypothetical protein |
| ECs0747 | 832205 | 832549 | succinate dehydrogenase hydrophobic subunit |
| ECs0749 | 834334 | 835047 | succinate dehydrogenase |
| ECs0751 | 835351 | 838149 | 2-oxoglutarate dehydrogenase decarboxylase component |
| ECs0752 | 838167 | 839381 | 2-oxoglutarate dehydrogenase dihydrolipoyltranssuccinase E2 component |
| ECs0753 | 839478 | 840641 | succinyl-CoA synthetase beta subunit |
| ECs0754 | 840644 | 841510 | succinyl-CoA synthetase alpha subunit |
| ECs0769 | 857548 | 858684 | cytochrome d terminal oxidase polypeptide subunit II |
| ECs0770 | 858815 | 859105 | hypothetical protein |
| ECs0771 | 859258 | 859659 | hypothetical protein |
| ECs0773 | 860355 | 860780 | putative inner membrane protein TolR |
| ECs0778 | 865949 | 866989 | quinolinate synthetase A protein |
| ECs0781 | 868804 | 869181 | putative homeobox protein |
| ECs0785 | 872706 | 873851 | galactokinase |
| ECs0789 | 877732 | 878517 | molybdate uptake regulatory protein |
| ECs0792 | 879735 | 880421 | molybdate transport permease protein |
| ECs0793 | 880427 | 881482 | ATP-binding component of molybdate transport system |
| ECs0795 | 882459 | 883451 | putative isomerase |
| ECs0851 | 929802 | 930275 | hypothetical protein |
| ECs0856 | 934636 | 935310 | dethiobiotin synthetase |
| ECs0860 | 940420 | 940929 | molybdenum cofactor biosynthesis protein B |
| ECs0861 | 940935 | 941417 | molybdenum cofactor biosynthesis protein C |
| ECs0862 | 941413 | 941655 | molybdopterin biosynthesis protein D chain |
| ECs0863 | 941660 | 942109 | molybdopterin biosynthesis protein E chain |
| ECs0867 | 944868 | 946106 | cardiolipin synthetase |
| ECs0868 | 946106 | 946864 | hypothetical protein |
| ECs0869 | 946997 | 947404 | hypothetical protein |
| ECs0870 | 947372 | 948475 | hypothetical protein |
| ECs0878 | 959688 | 960647 | putative enzyme |
| ECs0880 | 962108 | 962365 | hypothetical protein |
| ECs0881 | 962633 | 962896 | hypothetical protein |
| ECs0883 | 963703 | 965982 | putative outer membrane receptor for iron transport |
| ECs0886 | 967709 | 969931 | putative transport protein |
| ECs0887 | 970051 | 970770 | ATP-binding component of glutamine high-affinity transport system |
| ECs0888 | 970770 | 971426 | membrane component of glutamine high-affinity transport system |
| ECs0889 | 971567 | 972310 | permease of periplasmic glutamine-binding protein |
| ECs0893 | 975323 | 976903 | putative enzyme |
| ECs0894 | 977489 | 977953 | putative toxin |
| ECs0896 | 979132 | 980049 | hypothetical protein |
| ECs0903 | 987873 | 988532 | putative transaldolase |
| ECs0911 | 996224 | 997132 | putative transport system permease protein |
| ECs0915 | 1002001 | 1003323 | hypothetical protein |
| ECs0917 | 1004030 | 1005142 | putative dehydrogenase |
| ECs0920 | 1007266 | 1008021 | transcriptional repressor for deo operon, tsx, nupG |
| ECs0921 | 1008082 | 1008675 | hypothetical protein |
| ECs0928 | 1015376 | 1015750 | hypothetical protein |
| ECs0929 | 1015786 | 1016040 | glutaredoxin1 redox coenzyme for glutathione-dependent ribonucleotide reductase |
| ECs0930 | 1016200 | 1016484 | hypothetical protein |
| ECs0931 | 1016471 | 1017190 | modulator of drug activity A |
| ECs0932 | 1017254 | 1018153 | ribosomal protein S6 modification protein |
| ECs0938 | 1023276 | 1023761 | hypothetical protein |
| ECs0944 | 1029466 | 1030131 | arginine 3rd transport system permease protein |
| ECs0947 | 1031606 | 1032331 | ATP-binding component of 3rd arginine transport system |
| ECs0956 | 1039408 | 1040406 | putative arylsulfatase |
| ECs0960 | 1045073 | 1045969 | putative surface protein |
| ECs0962 | 1047548 | 1049203 | hypothetical protein |
| ECs0964 | 1050310 | 1051422 | putative membrane protein |
| ECs0967 | 1053988 | 1054305 | hypothetical protein |
| ECs0968 | 1054339 | 1056612 | ATP-binding component of serine protease |
| ECs0969 | 1057305 | 1057520 | protein chain initiation factor IF-1 |
| ECs0970 | 1057808 | 1058509 | leucyl, phenylalanyl-tRNA-protein transferase |
| ECs0971 | 1058554 | 1060272 | ATP-binding component of cytochrome-related transport |
| ECs0972 | 1060276 | 1062039 | ATP-binding component of cytochrome-related transport |
| ECs0977 | 1069106 | 1070446 | putative polynucleotide enzyme |
| ECs0978 | 1070540 | 1071829 | serine tRNA synthetase |
| ECs0983 | 1076985 | 1078130 | putative transport |
| ECs0989 | 1086405 | 1087094 | hypothetical protein |
| ECs0993 | 1090844 | 1091524 | cytidylate kinase |
| ECs0994 | 1091638 | 1093308 | 30S ribosomal subunit protein S1 |
| ECs0995 | 1093471 | 1093752 | integration host factor (IHF) beta subunit |
| ECs1000 | 1100315 | 1100494 | hypothetical protein |
| ECs1001 | 1100494 | 1101237 | CTP:CMP-3-deoxy-D-manno-octulosonate transferase |
| ECs1002 | 1101394 | 1102284 | hypothetical protein |
| ECs1003 | 1102267 | 1103043 | hypothetical protein |
| ECs1010 | 1113292 | 1113936 | hypothetical protein |
| ECs1011 | 1113993 | 1115180 | aspartate aminotransferase |
| ECs1018 | 1124318 | 1125460 | alkanesulfonate monooxygenase |
| ECs1034 | 1141472 | 1142722 | paraquat-inducible protein A |
| ECs1038 | 1145426 | 1145941 | beta-hydroxydecanoyl thioester dehydrase |
| ECs1039 | 1146013 | 1147770 | putative ATP-dependent protease |
| ECs1040 | 1147956 | 1148405 | putative dehydrogenase |
| ECs1045 | 1153376 | 1153819 | hypothetical protein |
| ECs1048 | 1156585 | 1157244 | hypothetical protein |
| ECs1053 | 1159813 | 1160139 | putative sulfite reductase |
| ECs1054 | 1160233 | 1160889 | putative carrier/transport protein |
| ECs1129 | 1212374 | 1214164 | hydrogenase-1 large subunit |
| ECs1130 | 1214186 | 1214890 | probable Ni/Fe-hydrogenase 1 b-type cytochrome subunit |
| ECs1132 | 1215474 | 1215869 | hydrogenase 1 formation factor HyaE |
| ECs1135 | 1218416 | 1219549 | probable third cytochrome oxidase subunit II |
| ECs1145 | 1229815 | 1230024 | cold shock-like protein CspG |
| ECs1147 | 1230645 | 1231715 | hypothetical protein |
| ECs1150 | 1235618 | 1236307 | response transcriptional regulator for torA |
| ECs1154 | 1240908 | 1241210 | hypothetical protein |
| ECs1159 | 1245220 | 1245444 | hypothetical protein |
| ECs1254 | 1311188 | 1311775 | putative enzyme |
| ECs1264 | 1325195 | 1326319 | hypothetical protein |
| ECs1412 | 1458780 | 1459331 | putative oxidoreductase component |
| ECs1415 | 1460854 | 1461267 | curli production assembly/transport component CsgF |
| ECs1416 | 1461295 | 1461681 | curli production assembly/transport component CsgE |
| ECs1417 | 1461689 | 1462336 | transcriptional regulator for 2nd curli operon |
| ECs1423 | 1464962 | 1465492 | putative polyprotein |
| ECs1434 | 1477045 | 1477617 | hypothetical protein |
| ECs1437 | 1478612 | 1479727 | sarcosine oxidase-like protein |
| ECs1442 | 1482554 | 1483198 | glutaredoxin 2 |
| ECs1443 | 1483265 | 1484470 | hypothetical protein |
| ECs1444 | 1484706 | 1485287 | ribosomal-protein-alanine N-acetyltransferase RimJ |
| ECs1445 | 1485301 | 1485945 | hypothetical protein |
| ECs1450 | 1489351 | 1490007 | flagella basal body P-ring formation protein FlgA |
| ECs1451 | 1490162 | 1490575 | flagellar rod protein FlgB |
| ECs1452 | 1490582 | 1490983 | flagellar rod protein FlgC |
| ECs1453 | 1490998 | 1491690 | flagellar biosynthesis FlgD protein |
| ECs1455 | 1492943 | 1493695 | flagellar rod protein flgF |
| ECs1456 | 1493836 | 1494615 | flagellar basal body rod protein flgG |
| ECs1459 | 1496478 | 1497416 | flagellar basal body protein FlgJ |
| ECs1460 | 1497485 | 1499125 | flagellar hook-filament junction protein 1 FlgK |
| ECs1461 | 1499140 | 1500090 | flagellar hook-filament junction protein 3 FlgL |
| ECs1464 | 1504046 | 1505002 | hypothetical protein |
| ECs1466 | 1505889 | 1506407 | hypothetical protein |
| ECs1469 | 1507854 | 1508804 | 3-oxoacyl-[acyl-carrier-protein] synthase III; acetylCoA ACP transacylase |
| ECs1470 | 1508823 | 1509749 | malonyl-CoA-[acyl-carrier-protein] transacylase |
| ECs1472 | 1510710 | 1510943 | acyl carrier protein |
| ECs1473 | 1511034 | 1512272 | 3-oxoacyl-[acyl-carrier-protein] synthase II |
| ECs1476 | 1514219 | 1514857 | thymidylate kinase |
| ECs1477 | 1514857 | 1515858 | DNA polymerase III delta prime subunit |
| ECs1479 | 1516964 | 1518394 | PTS system, glucose-specific IIBC component |
| ECs1482 | 1521354 | 1521728 | hypothetical protein |
| ECs1484 | 1522367 | 1523188 | putative beta-glucosidase |
| ECs1490 | 1528197 | 1528451 | hypothetical protein |
| ECs1494 | 1534552 | 1535748 | hypothetical protein |
| ECs1497 | 1537718 | 1538626 | putative NAGC-like transcriptional regulator |
| ECs1500 | 1540668 | 1541459 | spermidine/putrescine transport system permease |
| ECs1571 | 1590723 | 1591856 | ATP-binding component of spermidine/putrescine transport |
| ECs1601 | 1610044 | 1611501 | sensor protein PhoQ |
| ECs1602 | 1611504 | 1612172 | transcriptional regulatory protein |
| ECs1669 | 1666039 | 1666848 | cell division inhibitor MinD |
| ECs1670 | 1666875 | 1667567 | septum-site determining protein MinC |
| ECs1671 | 1668087 | 1668452 | hypothetical protein |
| ECs1675 | 1669565 | 1670221 | putative isomerase |
| ECs1678 | 1672253 | 1672669 | UmuD protein |
| ECs1682 | 1676425 | 1677141 | fatty acid metabolism regulator protein |
| ECs1683 | 1677199 | 1678728 | putative sporulation protein |
| ECs1688 | 1683697 | 1684608 | L, D-carboxyprptidase |
| ECs1708 | 1702606 | 1703694 | putative GTP-binding protein |
| ECs1709 | 1703814 | 1704395 | peptidyl-tRNA hydrolase |
| ECs1710 | 1704673 | 1704948 | hypothetical protein |
| ECs1716 | 1710894 | 1711973 | peptide chain release factor RF-1 |
| ECs1717 | 1711976 | 1712806 | possible protoporphyrinogen oxidase |
| ECs1718 | 1712806 | 1713195 | hypothetical protein |
| ECs1719 | 1713202 | 1714008 | hypothetical protein |
| ECs1720 | 1714047 | 1714898 | 2-dehydro-3-deoxyphosphooctulonate aldolase |
| ECs1722 | 1717461 | 1717688 | cation transport regulator |
| ECs1724 | 1718594 | 1718944 | hypothetical protein |
| ECs1730 | 1728827 | 1730362 | nitrate reductase 1 beta subunit |
| ECs1731 | 1730362 | 1731069 | nitrate reductase 1 delta subunit |
| ECs1732 | 1731072 | 1731746 | nitrate reductase 1 gamma subunit |
| ECs1734 | 1732830 | 1733669 | formyltetrahydrofolate deformylase |
| ECs1735 | 1733722 | 1734177 | hypothetical protein |
| ECs1737 | 1735287 | 1736297 | Hnr protein |
| ECs1738 | 1736502 | 1737407 | glucose-1-phosphate uridylyltransferase |
| ECs1739 | 1737557 | 1737967 | DNA-binding protein H-NS |
| ECs1740 | 1738571 | 1739185 | thymidine kinase |
| ECs1742 | 1742641 | 1743285 | putative channel protein |
| ECs1744 | 1745743 | 1746660 | oligopeptide transport permease protein |
| ECs1745 | 1746678 | 1747583 | oligopeptide transport system permease protein OppC |
| ECs1746 | 1747598 | 1748608 | oligopeptide ABC transport system ATP-binding protein OppD |
| ECs1747 | 1748608 | 1749609 | oligopeptide ABC transport system ATP-binding protein OppF |
| ECs1749 | 1750032 | 1751489 | cardiolipin synthase |
| ECs1751 | 1753417 | 1753710 | hypothetical protein |
| ECs1753 | 1754696 | 1755091 | hypothetical protein |
| ECs1754 | 1755199 | 1755735 | hypothetical protein |
| ECs1755 | 1755768 | 1756508 | hypothetical protein |
| ECs1833 | 1818245 | 1819435 | tryptophan synthase beta protein |
| ECs1843 | 1829064 | 1829819 | putative oxidoreductase |
| ECs1845 | 1831127 | 1831375 | hypothetical protein |
| ECs1847 | 1834562 | 1835533 | Positive transcriptional regulator for cysteine regulon |
| ECs1851 | 1840037 | 1840798 | non-essential phosphatidylglycerophosphate phosphatase |
| ECs1852 | 1840950 | 1841255 | hypothetical protein |
| ECs1853 | 1841265 | 1842431 | putative heat shock protein |
| ECs1856 | 1843819 | 1844034 | osmotically inducible lipoprotein |
| ECs1857 | 1844306 | 1845052 | putative DEOR-type transcriptional regulator |
| ECs1861 | 1850959 | 1851744 | enoyl-[acyl-carrier-protein] reductase (NADH) |
| ECs1868 | 1860572 | 1861561 | putative ATP-binding protein of peptide transport system |
| ECs1869 | 1861564 | 1862451 | homolog of Salmonella peptide transport permease protein |
| ECs1870 | 1862441 | 1863403 | homolog of Salmonella peptide transport permease protein |
| ECs1871 | 1863403 | 1865043 | homolog of Salmonella peptide transport periplasmic protein |
| ECs1872 | 1865359 | 1865601 | hypothetical protein |
| ECs1880 | 1874757 | 1875731 | psp operon transcriptional activator |
| ECs1881 | 1875898 | 1876563 | phage shock protein |
| ECs1882 | 1876620 | 1876841 | phage shock protein |
| ECs1883 | 1876844 | 1877200 | phage shock protein |
| ECs1884 | 1877212 | 1877430 | phage shock protein |
| ECs1885 | 1877508 | 1877819 | phage shock protein |
| ECs1901 | 1893325 | 1894383 | hypothetical protein |
| ECs1903 | 1896122 | 1896625 | thiol peroxidase |
| ECs1912 | 1904252 | 1905280 | hypothetical protein |
| ECs1914 | 1905834 | 1906781 | hypothetical protein |
| ECs1924 | 1915451 | 1916011 | hypothetical protein |
| ECs1926 | 1917522 | 1918502 | hypothetical protein |
| ECs2002 | 1979359 | 1980345 | fermentative D-lactate dehydrogenase |
| ECs2014 | 1998088 | 1998690 | acyl carrier protein phosphodiesterase |
| ECs2070 | 2056548 | 2058089 | cryptic nitrate reductase 2 beta subunit |
| ECs2072 | 2061911 | 2063296 | nitrite extrusion protein 2 |
| ECs2085 | 2077311 | 2077523 | hypothetical protein |
| ECs2086 | 2077868 | 2078296 | osmotically inducible protein |
| ECs2096 | 2088752 | 2090068 | hypothetical protein |
| ECs2097 | 2090202 | 2091734 | acid sensitivity protein |
| ECs2101 | 2098904 | 2100586 | putative ATP-binding component of a transport system |
| ECs2104 | 2104175 | 2104933 | putative ARAC-type regulatory protein |
| ECs2106 | 2105455 | 2107731 | putative oxidoreductase major subunit |
| ECs2131 | 2136739 | 2137662 | putative glutaminase |
| ECs2135 | 2141440 | 2142627 | L-arabinose and isopropyl-beta-D-thiogalactopyranoside export protein |
| ECs2137 | 2143532 | 2143963 | repressor of mar operon |
| ECs2138 | 2143986 | 2144366 | transcriptional activator of defense systems |
| ECs2149 | 2154248 | 2154931 | hypothetical protein |
| ECs2150 | 2155111 | 2155311 | hypothetical protein |
| ECs2289 | 2255611 | 2255949 | hypothetical protein |
| ECs2290 | 2255987 | 2256544 | spermidine N1-acetyltransferase |
| ECs2297 | 2264309 | 2264920 | putative oxidoreductase component |
| ECs2300 | 2267149 | 2268366 | putative NAGC-like transcriptional regulator |
| ECs2301 | 2268504 | 2269394 | putative transcriptional regulator LYSR-type |
| ECs2302 | 2269501 | 2270751 | putative transport protein |
| ECs2308 | 2274776 | 2276161 | pyridine nucleotide transhydrogenase beta subunit |
| ECs2310 | 2278228 | 2279169 | hypothetical protein |
| ECs2312 | 2280777 | 2281496 | putative oxidoreductase |
| ECs2313 | 2281499 | 2281831 | hypothetical protein |
| ECs2317 | 2284989 | 2286389 | fumarase C |
| ECs2323 | 2292512 | 2293882 | glucuronide permease |
| ECs2326 | 2296086 | 2296673 | repressor for uid operon |
| ECs2327 | 2296905 | 2297669 | NAD-dependent 7alpha-hydroxysteroid dehydrogenase |
| ECs2330 | 2300586 | 2301755 | putative aminotrasferase |
| ECs2331 | 2301862 | 2302860 | adenosine deaminase |
| ECs2334 | 2304580 | 2304792 | hypothetical protein |
| ECs2336 | 2305398 | 2305976 | hypothetical protein |
| ECs2337 | 2305979 | 2306554 | hypothetical protein |
| ECs2340 | 2309931 | 2310548 | hypothetical protein |
| ECs2341 | 2310555 | 2311247 | hypothetical protein |
| ECs2343 | 2312496 | 2313995 | putative transport protein |
| ECs2344 | 2314104 | 2314706 | glutathionine S-transferase |
| ECs2347 | 2317081 | 2317734 | pyridoxinephosphate oxidase |
| ECs2350 | 2319602 | 2320066 | putative outer membrane protein |
| ECs2359 | 2326385 | 2327479 | N-ethylmaleimide reductase |
| ECs2360 | 2327563 | 2327967 | lactoylglutathione lyase |
| ECs2361 | 2328073 | 2328717 | RNase T |
| ECs2363 | 2333483 | 2333827 | hypothetical protein |
| ECs2365 | 2335105 | 2335683 | superoxide dismutase |
| ECs2366 | 2335835 | 2337001 | putative transport protein |
| ECs2367 | 2337555 | 2338577 | transcriptional repressor for pur regulon |
| ECs2368 | 2338580 | 2339509 | putative transcriptional regulator LYSR-type |
| ECs2369 | 2339622 | 2340830 | putative transport protein |
| ECs2371 | 2342315 | 2342953 | riboflavin synthase alpha chain |
| ECs2374 | 2346411 | 2346713 | hypothetical protein |
| ECs2377 | 2349277 | 2350059 | hypothetical protein |
| ECs2381 | 2353565 | 2354188 | putative oxidoreductase Fe-S subunit |
| ECs2382 | 2354647 | 2354853 | hypothetical protein |
| ECs2384 | 2357133 | 2357366 | murein lipoprotein |
| ECs2386 | 2358588 | 2359001 | hypothetical protein |
| ECs2389 | 2361480 | 2362223 | putative ATP-binding component of a transport system |
| ECs2395 | 2368786 | 2369895 | hypothetical protein |
| ECs2399 | 2373501 | 2374364 | putative oxidoreductase |
| ECs2400 | 2374398 | 2375153 | 3-dehydroquinate dehydratase |
| ECs2404 | 2379331 | 2380092 | putative transport protein |
| ECs2405 | 2380115 | 2381050 | putative flavoprotein |
| ECs2406 | 2381109 | 2382395 | flavoprotein |
| ECs2407 | 2382395 | 2382685 | hypothetical protein |
| ECs2409 | 2384451 | 2386826 | phosphoenolpyruvate synthase |
| ECs2410 | 2387159 | 2387989 | hypothetical protein |
| ECs2411 | 2388149 | 2389192 | 3-deoxy-D-arabinoheptulosonate-7-phosphate synthase |
| ECs2414 | 2391024 | 2391734 | hypothetical protein |
| ECs2415 | 2391984 | 2392445 | lipoprotein |
| ECs2416 | 2392526 | 2393272 | ATP-binding component of vitamin B12 transport system |
| ECs2417 | 2393275 | 2393823 | vitamin B12 transport |
| ECs2418 | 2393889 | 2394866 | vitamin B12 transport permease protein |
| ECs2419 | 2394970 | 2395266 | integration host factor IHF alpha subunit |
| ECs2421 | 2397676 | 2398656 | phenylalanine tRNA synthetase alpha-subunit |
| ECs2423 | 2399109 | 2399462 | 50S ribosomal subunit protein L20 |
| ECs2426 | 2400358 | 2402283 | threonine tRNA synthetase |
| ECs2430 | 2407114 | 2407401 | hypothetical protein |
| ECs2433 | 2409094 | 2409759 | putative phosphatase |
| ECs2435 | 2410648 | 2412036 | part of a kinase |
| ECs2442 | 2418970 | 2419317 | PEP-dependent phosphotransferase enzyme III for cellobiose |
| ECs2444 | 2420814 | 2421131 | PEP-dependent phosphotransferase enzyme IV for cellobiose |
| ECs2445 | 2421434 | 2421769 | activator of ntrL gene |
| ECs2446 | 2421971 | 2422795 | NAD synthetase |
| ECs2447 | 2423028 | 2423912 | putative excinuclease subunit |
| ECs2449 | 2424656 | 2425138 | spheroplast protein Y |
| ECs2459 | 2435027 | 2435572 | hypothetical protein |
| ECs2465 | 2441002 | 2441406 | hypothetical protein |
| ECs2474 | 2451163 | 2452176 | cytoplasmic L-asparaginase I |
| ECs2475 | 2452190 | 2452828 | hypothetical protein |
| ECs2491 | 2467334 | 2468077 | putative scaffolding protein in the formation of a murein-synthesizing holoenzyme |
| ECs2493 | 2470560 | 2471840 | hypothetical protein |
| ECs2496 | 2475179 | 2475679 | hypothetical protein |
| ECs2498 | 2475957 | 2476400 | hypothetical protein |
| ECs2500 | 2477278 | 2478456 | putative amino acid/amine transport protein |
| ECs2507 | 2481728 | 2482363 | hypothetical protein |
| ECs2515 | 2491783 | 2492361 | putative outer membrane protein |
| ECs2516 | 2492404 | 2493096 | hypothetical protein |
| ECs2517 | 2493157 | 2495064 | putative enzyme |
| ECs2520 | 2496384 | 2496560 | hypothetical protein |
| ECs2522 | 2497999 | 2498574 | hypothetical protein |
| ECs2523 | 2498761 | 2500122 | L-serine deaminase |
| ECs2527 | 2503877 | 2504845 | mannose-specific PTS enzyme IIAB |
| ECs2528 | 2504911 | 2505708 | mannose-specific PTS enzyme IIC |
| ECs2532 | 2508084 | 2508890 | putative enzyme |
| ECs2533 | 2509059 | 2509265 | cold shock protein |
| ECs2534 | 2509281 | 2509421 | hypothetical protein |
| ECs2535 | 2510093 | 2510377 | hypothetical protein |
| ECs2539 | 2513528 | 2514406 | heat shock protein HtpX |
| ECs2541 | 2516669 | 2517364 | ProP effector |
| ECs2550 | 2525553 | 2526422 | putative resistance protein |
| ECs2551 | 2526429 | 2526800 | hypothetical protein |
| ECs2553 | 2527271 | 2527924 | hypothetical protein |
| ECs2556 | 2530882 | 2531538 | hypothetical protein |
| ECs2558 | 2532291 | 2532578 | hypothetical protein |
| ECs2560 | 2533949 | 2534587 | 2-keto-3-deoxygluconate 6-phosphate aldolase/2-keto-4-hydroxyglutarate aldolase |
| ECs2562 | 2536673 | 2538145 | glucose-6-phosphate dehydrogenase |
| ECs2563 | 2538483 | 2539349 | hypothetical protein |
| ECs2565 | 2541056 | 2542024 | heat shock protein MsbB |
| ECs2566 | 2542147 | 2543466 | hypothetical protein |
| ECs2568 | 2544493 | 2545245 | putative ATP-binding component of a transport system |
| ECs2569 | 2545245 | 2546027 | hypothetical protein |
| ECs2571 | 2547298 | 2547906 | Holliday junction helicase subunit B |
| ECs2574 | 2549344 | 2550081 | hypothetical protein |
| ECs2576 | 2550683 | 2552452 | aspartate tRNA synthetase |
| ECs2580 | 2554632 | 2555372 | hypothetical protein |
| ECs2583 | 2558965 | 2560062 | putative cytochrome C-type protein |
| ECs2584 | 2560453 | 2561196 | copper homeostasis protein |
| ECs2586 | 2561992 | 2563722 | arginine tRNA synthetase |
| ECs2591 | 2568326 | 2568967 | CheY protein phophatase |
| ECs2592 | 2568981 | 2569367 | chemotaxis protein CheY |
| ECs2593 | 2569385 | 2570431 | response regulator for chemotaxis CheA sensor |
| ECs2594 | 2570437 | 2571294 | response regulator for chemotaxis |
| ECs2597 | 2574767 | 2575267 | Positive regulator of CheA protein activity |
| ECs2599 | 2577260 | 2578183 | chemotaxis protein MotB |
| ECs2600 | 2578183 | 2579067 | proton conductor component of motor |
| ECs2603 | 2580905 | 2581330 | putative regulator |
| ECs2608 | 2584710 | 2586221 | ATP-binding component of high-affinity L-arabinose transport system |
| ECs2610 | 2588077 | 2588577 | ferritin-like protein |
| ECs2612 | 2589374 | 2589694 | hypothetical protein |
| ECs2614 | 2590405 | 2590641 | hypothetical protein |
| ECs2615 | 2590833 | 2592041 | tyrosine-specific transport system |
| ECs2616 | 2592109 | 2592771 | hypothetical protein |
| ECs2650 | 2614543 | 2615088 | phosphatidylglycerophosphate synthetase |
| ECs2669 | 2632000 | 2632230 | hypothetical protein |
| ECs2681 | 2642492 | 2642932 | flagellar fliJ protein |
| ECs2682 | 2642932 | 2644056 | flagellar hook-length control protein |
| ECs2683 | 2644164 | 2644625 | flagellar protein FliL |
| ECs2687 | 2646415 | 2647149 | flagellar biosynthetic protein FliP |
| ECs2688 | 2647162 | 2647428 | putative export protein FliQ |
| ECs2690 | 2648514 | 2649134 | transcriptional regulator for ctr capsule biosynthesis |
| ECs2691 | 2649184 | 2649369 | hypothetical protein |
| ECs2693 | 2650057 | 2650869 | hypothetical protein |
| ECs2697 | 2654085 | 2655002 | putative transmembrane subunit |
| ECs2699 | 2655448 | 2656863 | DNA cytosine methylase |
| ECs2708 | 2663495 | 2663905 | hypothetical protein |
| ECs2710 | 2665021 | 2665653 | hypothetical protein |
| ECs3034 | 2966013 | 2966705 | putative seritonin transporter |
| ECs3035 | 2966838 | 2967719 | cytidine/deoxycytidine deaminase |
| ECs3036 | 2967872 | 2968588 | vancomycin sensitivity |
| ECs3037 | 2968594 | 2968830 | hypothetical protein |
| ECs3040 | 2971740 | 2972747 | methyl-galactoside transport system permease protein |
| ECs3041 | 2972766 | 2974283 | ATP-binding component of methyl-galactoside transport system |
| ECs3042 | 2974347 | 2975342 | galactose-binding transport protein |
| ECs3045 | 2977980 | 2978645 | GTP cyclohydrolase I |
| ECs3048 | 2982045 | 2983511 | lysine-specific permease |
| ECs3049 | 2983719 | 2984597 | putative transcriptional regulator LYSR-type |
| ECs3050 | 2984696 | 2985742 | hypothetical protein |
| ECs3051 | 2985819 | 2986673 | endonuclease IV |
| ECs3061 | 2997297 | 2998424 | fructose-specific PTS system IIA component |
| ECs3067 | 3004909 | 3005472 | putative lipoprotein |
| ECs3070 | 3009109 | 3010200 | putative transport system permease protein |
| ECs3071 | 3010203 | 3011225 | putative transport system permease protein |
| ECs3072 | 3011230 | 3012816 | putative ATP-binding component of a transport system |
| ECs3073 | 3012826 | 3013167 | hypothetical protein |
| ECs3075 | 3014721 | 3015413 | 16S pseudouridylate 516 synthase |
| ECs3076 | 3015562 | 3017319 | putative ATP-dependent helicase |
| ECs3077 | 3017447 | 3017728 | 50S ribosomal subunit protein L25 |
| ECs3078 | 3017873 | 3018877 | nucleoid-associated protein |
| ECs3079 | 3019059 | 3019283 | hypothetical protein |
| ECs3085 | 3026517 | 3028457 | cytochrome c-type biogenesis protein |
| ECs3086 | 3028457 | 3028933 | cytochrome-c biosynthesis heme-carrier protein ccmE |
| ECs3089 | 3029918 | 3030577 | heme exporter protein B |
| ECs3091 | 3031213 | 3031812 | cytochrome c-type protein |
| ECs3092 | 3031825 | 3032271 | cytochrome c-type protein |
| ECs3093 | 3032271 | 3033131 | ferredoxin-type protein |
| ECs3094 | 3033121 | 3033813 | ferredoxin-type protein |
| ECs3095 | 3033823 | 3036306 | probable nitrate reductase 3 |
| ECs3096 | 3036306 | 3036566 | hypothetical protein |
| ECs3114 | 3062566 | 3065190 | DNA gyrase subunit A |
| ECs3117 | 3070648 | 3072930 | ribonucleoside diphosphate reductase 1 alpha subunit |
| ECs3119 | 3074290 | 3074541 | hypothetical protein |
| ECs3120 | 3074601 | 3075248 | InaA protein |
| ECs3128 | 3084893 | 3086080 | anaerobic sn-glycerol-3-phosphate dehydrogenase K-small subunit |
| ECs3142 | 3097584 | 3098549 | putative sugar transferase |
| ECs3145 | 3101421 | 3103070 | hypothetical protein |
| ECs3150 | 3106478 | 3107332 | dihydroxynaphtoic acid synthetase |
| ECs3153 | 3109864 | 3111156 | isochorismate hydroxymutase 2 |
| ECs3154 | 3111238 | 3111540 | hypothetical protein |
| ECs3159 | 3115581 | 3116081 | hypothetical protein |
| ECs3162 | 3119378 | 3121216 | NADH dehydrogenase I chain L |
| ECs3163 | 3121216 | 3121515 | NADH dehydrogenase I chain K |
| ECs3164 | 3121515 | 3122066 | NADH dehydrogenase I chain J |
| ECs3165 | 3122081 | 3122620 | NADH dehydrogenase I chain I |
| ECs3166 | 3122638 | 3123612 | NADH dehydrogenase I chain H |
| ECs3169 | 3127725 | 3128222 | NADH dehydrogenase I chain E |
| ECs3171 | 3130124 | 3130783 | NADH dehydrogenase I chain B |
| ECs3174 | 3133731 | 3134945 | putative aminotransferase |
| ECs3175 | 3135032 | 3135628 | putative alpha helix protein |
| ECs3176 | 3135693 | 3137522 | putative transport protein |
| ECs3177 | 3137612 | 3138259 | putative phosphatase |
| ECs3180 | 3139641 | 3140840 | acetate kinase |
| ECs3181 | 3140918 | 3143059 | phosphotransacetylase |
| ECs3183 | 3144808 | 3145347 | putative regulator |
| ECs3186 | 3146792 | 3147436 | putative S-transferase |
| ECs3187 | 3147496 | 3147855 | D-erythro-7,8-dihydroneopterin tri P epimerase |
| ECs3190 | 3149910 | 3150680 | ATP-binding component of histidine transport |
| ECs3193 | 3152180 | 3152959 | histidine transport system histidine-binding periplasmic protein |
| ECs3195 | 3154231 | 3154797 | 3-octaprenyl-4-hydroxybenzoate carboxy-lyase |
| ECs3196 | 3154895 | 3156409 | amidophosphoribosyltransferase |
| ECs3197 | 3156449 | 3156934 | membrane protein required for colicin V production |
| ECs3198 | 3157378 | 3158037 | putative lipoprotein |
| ECs3200 | 3159368 | 3160279 | acetylCoA carboxylase carboxytransferase component beta subunit |
| ECs3202 | 3161180 | 3161989 | pseudouridylate synthase I |
| ECs3207 | 3166751 | 3167968 | 3-oxoacyl-[acyl-carrier-protein] synthase I |
| ECs3209 | 3170257 | 3170532 | hypothetical protein |
| ECs3229 | 3190854 | 3191606 | lipoprotein precursor |
| ECs3230 | 3191900 | 3192829 | putative transport |
| ECs3246 | 3208778 | 3210313 | multidrug resistance protein Y |
| ECs3248 | 3211892 | 3212503 | putative Positive transcription regulator |
| ECs3252 | 3217378 | 3218319 | putative receptor protein |
| ECs3253 | 3218392 | 3220083 | putative enzyme |
| ECs3254 | 3220140 | 3221387 | putative enzyme |
| ECs3257 | 3223186 | 3223425 | hypothetical protein |
| ECs3262 | 3229264 | 3230118 | putative ARAC-type regulatory protein |
| ECs3266 | 3234784 | 3236028 | putative transport protein |
| ECs3268 | 3236598 | 3237560 | glucokinase |
| ECs3270 | 3239135 | 3239458 | hypothetical protein |
| ECs3271 | 3239604 | 3240839 | high affinity manganese transport protein |
| ECs3278 | 3247790 | 3249202 | glutamate tRNA synthetase catalytic subunit |
| ECs3282 | 3251325 | 3252320 | putative cytochrome oxidase |
| ECs3285 | 3255854 | 3256612 | required for sulfate transport |
| ECs3287 | 3258155 | 3258409 | PTS system protein HPr |
| ECs3289 | 3260225 | 3260731 | glucose-specific PTS system IIA component |
| ECs3290 | 3260780 | 3261628 | pyridoxal/pyridoxine/pyridoxamine kinase |
| ECs3294 | 3264238 | 3265110 | sulfate transport system permease W protein |
| ECs3300 | 3269958 | 3271379 | putative PTS enzyme II |
| ECs3308 | 3277236 | 3278285 | putative ARAC-type regulatory protein |
| ECs3316 | 3285700 | 3286533 | ethanolamine utilization protein EutJ |
| ECs3318 | 3287962 | 3288246 | detox protein |
| ECs3320 | 3288688 | 3289701 | ethanolamine utilization protein EutI |
| ECs3325 | 3292225 | 3294501 | putative multimodular enzyme |
| ECs3326 | 3294790 | 3295737 | transaldolase A |
| ECs3328 | 3297861 | 3298901 | hypothetical protein |
| ECs3330 | 3299673 | 3301649 | putative oxidoreductase Fe-S subunit |
| ECs3333 | 3307371 | 3307724 | hypothetical protein |
| ECs3334 | 3307731 | 3308855 | N-succinyl-diaminopimelate deacylase |
| ECs3335 | 3309170 | 3309865 | hypothetical protein |
| ECs3337 | 3311972 | 3312832 | hypothetical protein |
| ECs3342 | 3316573 | 3317040 | bacterioferritin comigratory protein |
| ECs3362 | 3338377 | 3339012 | phosphoribosylglycinamide formyltransferase 1 |
| ECs3364 | 3341257 | 3342795 | exopolyphosphatase |
| ECs3369 | 3347103 | 3348677 | GMP synthetase |
| ECs3374 | 3353618 | 3354793 | putative dehydrogenase |
| ECs3375 | 3354807 | 3355424 | hypothetical protein |
| ECs3376 | 3355445 | 3356716 | histidine tRNA synthetase |
| ECs3377 | 3356830 | 3357945 | hypothetical protein |
| ECs3380 | 3360577 | 3361005 | nucleoside diphosphate kinase |
| ECs3387 | 3373466 | 3374308 | putative thiosulfate sulfurtransferase |
| ECs3391 | 3377406 | 3377738 | [2FE-2S] ferredoxin |
| ECs3395 | 3380561 | 3380944 | NifU-like protein |
| ECs3397 | 3382301 | 3382786 | hypothetical protein |
| ECs3399 | 3383943 | 3384743 | extragenic suppressor protein SuhB |
| ECs3401 | 3385936 | 3387213 | hypothetical protein |
| ECs3419 | 3407439 | 3407774 | regulatory protein P-II for glutamine synthetase |
| ECs3427 | 3419142 | 3419987 | hypothetical protein |
| ECs3431 | 3422128 | 3422853 | RecO protein |
| ECs3432 | 3422868 | 3423770 | GTP-binding protein |
| ECs3435 | 3425713 | 3427509 | GTP-binding elongation factor |
| ECs3445 | 3436266 | 3436646 | putative formate acetyltransferase |
| ECs3448 | 3438933 | 3439349 | putative thioredoxin-like protein |
| ECs3457 | 3455153 | 3456130 | suppressor of ftsH mutation |
| ECs3458 | 3456265 | 3456999 | hypothetical protein |
| ECs3462 | 3457864 | 3459021 | chorismate mutase-P / prephenate dehydratase |
| ECs3466 | 3461994 | 3462509 | hypothetical protein |
| ECs3469 | 3464306 | 3464650 | 50S ribosomal subunit protein L19 |
| ECs3470 | 3464695 | 3465459 | tRNA (guanine-7-)-methyltransferase |
| ECs3472 | 3466060 | 3466305 | 30S ribosomal subunit protein S16 |
| ECs3473 | 3466445 | 3467803 | signal recognition particle protein Ffh |
| ECs3482 | 3474905 | 3475384 | small protein B |
| ECs3522 | 3513460 | 3514905 | succinate-semialdehyde dehydrogenase |
| ECs3524 | 3516440 | 3517837 | transport permease protein of gamma-aminobutyrate |
| ECs3525 | 3517861 | 3518520 | putative transcriptional regulator |
| ECs3526 | 3518527 | 3518973 | hypothetical protein |
| ECs3527 | 3519060 | 3519215 | hypothetical protein |
| ECs3528 | 3519398 | 3519694 | hypothetical protein |
| ECs3530 | 3520281 | 3520682 | DNA-binding protein |
| ECs3531 | 3521350 | 3521796 | hypothetical protein |
| ECs3532 | 3521839 | 3522180 | hypothetical protein |
| ECs3540 | 3528967 | 3530166 | ATP-binding component of glycine betaine / proline transport system |
| ECs3541 | 3530162 | 3531223 | high-affinity glycine betaine / proline transport system protein |
| ECs3542 | 3531283 | 3532272 | high-affinity glycine betaine / proline transport system protein |
| ECs3544 | 3533775 | 3534509 | hypothetical protein |
| ECs3545 | 3534502 | 3534834 | hypothetical protein |
| ECs3546 | 3534928 | 3535455 | regulator of plasmid mcrB operon |
| ECs3549 | 3538379 | 3538891 | autoinducer production protein LuxSE.c. |
| ECs3550 | 3539044 | 3540597 | gamma-glutamate-cysteine ligase |
| ECs3552 | 3541098 | 3541661 | putative phosphatase |
| ECs3553 | 3542892 | 3543074 | carbon storage regulator |
| ECs3555 | 3546070 | 3546567 | regulatory protein OraA |
| ECs3556 | 3546638 | 3547696 | RecA protein |
| ECs3567 | 3557631 | 3558761 | putative oxidoreductase |
| ECs3574 | 3566723 | 3567130 | formate hydrogenlyase maturation protein |
| ECs3576 | 3567897 | 3568436 | formate hydrogenlyase subunit-7 component F |
| ECs3580 | 3572925 | 3573533 | formate hydrogenlyase subunit-7 component B |
| ECs3581 | 3573661 | 3574119 | transcriptional repressor of hyc and hyp operons |
| ECs3583 | 3574685 | 3575554 | hydrogenase isoenzyme HypB |
| ECs3584 | 3575548 | 3575817 | hydrogenase isoenzyme HypC |
| ECs3585 | 3575820 | 3576938 | hydrogenase isoenzyme HypD |
| ECs3599 | 3590781 | 3591827 | putative hydrogenase subunit |
| ECs3600 | 3591827 | 3592303 | 2C-methyl-D-erythritol 2,4-cyclodiphosphate synthase |
| ECs3602 | 3593035 | 3593343 | hypothetical protein |
| ECs3606 | 3595947 | 3596852 | ATP:sulfurylase subunit 2 |
| ECs3633 | 3624558 | 3625226 | hypothetical protein |
| ECs3639 | 3629045 | 3630340 | enolase |
| ECs3640 | 3630431 | 3632065 | CTP synthetase |
| ECs3649 | 3643151 | 3644500 | putative transport protein |
| ECs3650 | 3644938 | 3645384 | hypothetical protein |
| ECs3651 | 3645405 | 3646184 | hypothetical protein |
| ECs3652 | 3646187 | 3646513 | hypothetical protein |
| ECs3653 | 3647138 | 3647680 | Syd protein |
| ECs3654 | 3647748 | 3648593 | hypothetical protein |
| ECs3655 | 3648708 | 3650069 | hypothetical protein |
| ECs3656 | 3650629 | 3651915 | probable serine transporter |
| ECs3660 | 3655444 | 3656088 | L-fuculose-1-phosphate aldolase |
| ECs3661 | 3656635 | 3657948 | fucose permease |
| ECs3662 | 3657984 | 3659756 | L-fucose isomerase |
| ECs3664 | 3661288 | 3661707 | protein of fucose operon |
| ECs3668 | 3664053 | 3664967 | Positive regulator of gcv operon |
| ECs3671 | 3666942 | 3667382 | hypothetical protein |
| ECs3672 | 3667439 | 3668242 | putative enzyme |
| ECs3675 | 3671642 | 3672970 | N-acetylglutamate synthase |
| ECs3681 | 3685152 | 3685556 | hypothetical protein |
| ECs3683 | 3686110 | 3686577 | prepilin peptidase dependent protein A |
| ECs3684 | 3686764 | 3687555 | thymidylate synthetase |
| ECs3685 | 3687565 | 3688437 | phosphatidylglycerol-prolipoprotein diacylglyceryl transferase |
| ECs3686 | 3688591 | 3690834 | PTS system transcriptional regulator enzyme I |
| ECs3687 | 3690850 | 3691377 | putative invasion protein |
| ECs3690 | 3693671 | 3693886 | hypothetical protein |
| ECs3691 | 3693997 | 3695034 | hypothetical protein |
| ECs3694 | 3699000 | 3700028 | repressor of galETK operon |
| ECs3699 | 3704901 | 3705659 | 2-deoxy-D-gluconate 3-dehydrogenase |
| ECs3700 | 3705692 | 3706525 | putative 5-keto 4-deoxyuronate isomerase |
| ECs3744 | 3745841 | 3747034 | putative dehydratase |
| ECs3745 | 3747095 | 3748303 | putative deacetylase |
| ECs3747 | 3749792 | 3750721 | putative kinase |
| ECs3753 | 3758690 | 3759466 | hypothetical protein |
| ECs3762 | 3771809 | 3773323 | lysine tRNA synthetase |
| ECs3766 | 3776999 | 3777892 | site-specific recombinase |
| ECs3767 | 3778004 | 3778522 | flavodoxin 2 |
| ECs3768 | 3778568 | 3778972 | hypothetical protein |
| ECs3769 | 3778956 | 3779219 | hypothetical protein |
| ECs3770 | 3779462 | 3780439 | hypothetical protein |
| ECs3772 | 3781345 | 3781653 | hypothetical protein |
| ECs3777 | 3788246 | 3789445 | hypothetical protein |
| ECs3779 | 3790647 | 3791969 | proline aminopeptidase P II |
| ECs3781 | 3792741 | 3793067 | hypothetical protein |
| ECs3784 | 3794703 | 3795932 | D-3-phosphoglycerate dehydrogenase |
| ECs3786 | 3797229 | 3798119 | replication initiation inhibitor |
| ECs3808 | 3818085 | 3819470 | mannitol-specific PTS system enzyme II component |
| ECs3810 | 3820258 | 3822246 | transketolase 1 isozyme |
| ECs3812 | 3823491 | 3824408 | agmatinase |
| ECs3818 | 3828192 | 3829343 | methionine adenosyltransferase 1 |
| ECs3825 | 3834999 | 3835412 | hypothetical protein |
| ECs3826 | 3836604 | 3837305 | hypothetical protein |
| ECs3828 | 3837326 | 3837889 | putative resistance protein |
| ECs3830 | 3838187 | 3838777 | putative ribosomal protein |
| ECs3833 | 3841279 | 3842322 | periplasmic L-asparaginase II |
| ECs3835 | 3843404 | 3843727 | hypothetical protein |
| ECs3836 | 3843730 | 3844446 | hypothetical protein |
| ECs3838 | 3845687 | 3845959 | hypothetical protein |
| ECs3875 | 3880073 | 3880318 | hydrogenase-2 operon protein HybG |
| ECs3876 | 3880334 | 3880672 | hydrogenase-2 operon protein HybF |
| ECs3877 | 3880668 | 3881153 | hydrogenase-2 operon protein HybE |
| ECs3878 | 3881149 | 3881640 | hydrogenase 2 maturation protease HybD |
| ECs3879 | 3881643 | 3883343 | hydrogenase-2 large subunit HybC |
| ECs3882 | 3885500 | 3886615 | putative hydrogenase subunit |
| ECs3883 | 3886807 | 3887091 | hypothetical protein |
| ECs3887 | 3890019 | 3890900 | putative oxidoreductase |
| ECs3889 | 3891178 | 3891600 | uptake of enterochelin; tonB-dependent uptake of B colicins |
| ECs3893 | 3893920 | 3894576 | hypothetical protein |
| ECs3894 | 3895712 | 3896872 | putative oxidoreductase |
| ECs3896 | 3896980 | 3897804 | 2,5-diketo-D-gluconate reductase |
| ECs3899 | 3898978 | 3899232 | hypothetical protein |
| ECs3901 | 3901611 | 3903020 | suppressor of ftsI |
| ECs3902 | 3903098 | 3903832 | 1-acyl-sn-glycerol-3-phosphate acyltransferase |
| ECs3903 | 3904069 | 3906324 | DNA topoisomerase IV subunit A |
| ECs3919 | 3921522 | 3922100 | hypothetical protein |
| ECs3920 | 3922103 | 3922927 | regulator of lacZ |
| ECs3921 | 3922955 | 3923374 | putative enzyme |
| ECs3922 | 3923378 | 3924004 | hypothetical protein |
| ECs3926 | 3926515 | 3927672 | putative synthetase/amidase |
| ECs3935 | 3934851 | 3936281 | ADP-heptose synthase |
| ECs3937 | 3939195 | 3940493 | hypothetical protein |
| ECs3945 | 3946901 | 3947503 | L-tartrate dehydratase subunit B |
| ECs3946 | 3947555 | 3949015 | hypothetical protein |
| ECs3948 | 3950312 | 3950524 | 30S ribosomal subunit protein S21 |
| ECs3953 | 3956310 | 3956930 | hypothetical protein |
| ECs3954 | 3957090 | 3958607 | aerotaxis sensor receptor |
| ECs3956 | 3960449 | 3960778 | putative tRNA synthetase |
| ECs3959 | 3965253 | 3965699 | evolved beta-D-galactosidase beta subunit |
| ECs3966 | 3974373 | 3975506 | putative enzyme |
| ECs3972 | 3980828 | 3981376 | hypothetical protein |
| ECs3977 | 3987405 | 3988064 | hypothetical protein |
| ECs3978 | 3988071 | 3988451 | hypothetical protein |
| ECs3980 | 3989007 | 3989309 | hypothetical protein |
| ECs3981 | 3989315 | 3989716 | hypothetical protein |
| ECs3982 | 3989709 | 3990005 | hypothetical protein |
| ECs3987 | 3992950 | 3993843 | putative transcriptional regulator LYSR-type |
| ECs3997 | 4004671 | 4005657 | threonine dehydratase |
| ECs3998 | 4005759 | 4006694 | transcriptional activator of tdc operon |
| ECs4004 | 4012025 | 4012792 | hypothetical protein |
| ECs4016 | 4023516 | 4024667 | putative tagatose-6-phosphate aldose/ketose isomerase |
| ECs4017 | 4024683 | 4025540 | tagatose-1,6-bisphosphate aldolase |
| ECs4018 | 4025710 | 4026183 | galactosamine-specific PTS system enzyme IIB component |
| ECs4029 | 4037892 | 4038284 | hypothetical protein |
| ECs4030 | 4038307 | 4038894 | hypothetical protein |
| ECs4037 | 4042756 | 4043256 | hypothetical protein |
| ECs4044 | 4050540 | 4051421 | lipoprotein precursor |
| ECs4046 | 4053915 | 4054181 | 30S ribosomal subunit protein S15 |
| ECs4047 | 4054333 | 4055274 | tRNA pseudouridine 5S synthase |
| ECs4048 | 4055277 | 4055675 | ribosome-binding factor A |
| ECs4052 | 4061135 | 4062475 | argininosuccinate synthetase |
| ECs4054 | 4064674 | 4065003 | protein-export protein SecG |
| ECs4055 | 4065234 | 4066568 | putative phosphoglucomutase / phosphomannomutase |
| ECs4056 | 4066564 | 4067409 | 7,8-dihydropteroate synthase |
| ECs4058 | 4069536 | 4070162 | cell division protein methyltransferase FtsJ |
| ECs4059 | 4070288 | 4070578 | hypothetical protein |
| ECs4060 | 4070740 | 4071213 | transcription elongation factor GreA |
| ECs4061 | 4071461 | 4072891 | D-alanyl-D-alanine carboxypeptidase |
| ECs4063 | 4074125 | 4075087 | hypothetical protein |
| ECs4064 | 4075217 | 4075471 | 50S ribosomal subunit protein L27 |
| ECs4066 | 4076062 | 4077030 | octaprenyl diphosphate synthase |
| ECs4067 | 4077262 | 4077537 | regulatory factor of maltose metabolism |
| ECs4068 | 4077591 | 4078847 | UDP-N-glucosamine 1-carboxyvinyltransferase |
| ECs4071 | 4079612 | 4080244 | hypothetical protein |
| ECs4073 | 4080822 | 4081601 | hypothetical protein |
| ECs4074 | 4081612 | 4082418 | putative ATP-binding component of a transport system |
| ECs4077 | 4084626 | 4085189 | hypothetical protein |
| ECs4078 | 4085189 | 4085761 | hypothetical protein |
| ECs4080 | 4086297 | 4087019 | putative ATP-binding component of a transport system |
| ECs4081 | 4087070 | 4088500 | RNA polymerase sigma(54 or 60) factor RpoN |
| ECs4082 | 4088526 | 4088810 | probable sigma-54 modulation protein |
| ECs4084 | 4089468 | 4090319 | hypothetical protein |
| ECs4085 | 4090319 | 4090588 | phosphocarrier protein HPr-like NPr |
| ECs4086 | 4090805 | 4091434 | hypothetical protein |
| ECs4087 | 4091437 | 4092162 | putative peptidoglycan enzyme |
| ECs4092 | 4101550 | 4102965 | glutamate synthase small subunit |
| ECs4098 | 4107933 | 4108823 | N-acetylneuraminate lyase |
| ECs4101 | 4111518 | 4112012 | stringent starvation protein B |
| ECs4102 | 4112021 | 4112656 | stringent starvation protein A |
| ECs4103 | 4113054 | 4113443 | 30S ribosomal subunit protein S9 |
| ECs4104 | 4113462 | 4113887 | 50S ribosomal subunit protein L13 |
| ECs4110 | 4119940 | 4120407 | repressor of arg regulon |
| ECs4112 | 4121097 | 4121366 | hypothetical protein |
| ECs4113 | 4121461 | 4123425 | hypothetical protein |
| ECs4116 | 4124754 | 4125680 | putative transcriptional regulator LYSR-type |
| ECs4117 | 4125820 | 4127262 | suppresses inhibitory activity of CsrA |
| ECs4119 | 4131289 | 4132755 | ribonuclease G |
| ECs4120 | 4132748 | 4133338 | hypothetical protein |
| ECs4121 | 4133350 | 4133835 | rod shape-determining protein |
| ECs4124 | 4136355 | 4138292 | hypothetical protein |
| ECs4125 | 4138444 | 4139415 | putative dehydrogenase |
| ECs4127 | 4140396 | 4140863 | acetylCoA carboxylase BCCP subunit |
| ECs4128 | 4140877 | 4142223 | acetyl CoA carboxylase biotin carboxylase subunit |
| ECs4132 | 4145240 | 4146202 | putative dehydrogenase |
| ECs4133 | 4146231 | 4146524 | site-specific DNA inversion stimulation factor |
| ECs4136 | 4147765 | 4148424 | putative transcriptional regulator |
| ECs4137 | 4148823 | 4149977 | transmembrane protein affects septum formation and cell membrane permeability |
| ECs4140 | 4153394 | 4153612 | hypothetical protein |
| ECs4150 | 4167006 | 4167476 | hypothetical protein |
| ECs4152 | 4168702 | 4169208 | peptide deformylase |
| ECs4153 | 4169226 | 4170170 | 10-formyltetrahydrofolate:L-methionyl-tRNA(fMet) N-formyltransferase |
| ECs4155 | 4171530 | 4172903 | transport of potassium |
| ECs4159 | 4174631 | 4175011 | 50S ribosomal subunit protein L17 |
| ECs4160 | 4175055 | 4176041 | RNA polymerase, alpha subunit |
| ECs4161 | 4176070 | 4176687 | 30S ribosomal subunit protein S4 |
| ECs4162 | 4176724 | 4177110 | 30S ribosomal subunit protein S11 |
| ECs4163 | 4177130 | 4177483 | 30S ribosomal subunit protein S13 |
| ECs4165 | 4177781 | 4179109 | putative ATPase subunit of translocase |
| ECs4166 | 4179120 | 4179551 | 50S ribosomal subunit protein L15 |
| ECs4167 | 4179558 | 4179734 | 50S ribosomal subunit protein L30 |
| ECs4168 | 4179741 | 4180241 | 30S ribosomal subunit protein S5 |
| ECs4169 | 4180259 | 4180609 | 50S ribosomal subunit protein L18 |
| ECs4170 | 4180622 | 4181152 | 50S ribosomal subunit protein L6 |
| ECs4171 | 4181168 | 4181557 | 30S ribosomal subunit protein S8 |
| ECs4172 | 4181594 | 4181896 | 30S ribosomal subunit protein S14 |
| ECs4173 | 4181914 | 4182450 | 50S ribosomal subunit protein L5 |
| ECs4174 | 4182468 | 4182779 | 50S ribosomal subunit protein L24 |
| ECs4175 | 4182793 | 4183161 | 50S ribosomal subunit protein L14 |
| ECs4176 | 4183329 | 4183580 | 30S ribosomal subunit protein S17 |
| ECs4177 | 4183583 | 4183771 | 50S ribosomal subunit protein L29 |
| ECs4178 | 4183774 | 4184181 | 50S ribosomal subunit protein L16 |
| ECs4179 | 4184197 | 4184895 | 30S ribosomal subunit protein S3 |
| ECs4180 | 4184916 | 4185245 | 50S ribosomal subunit protein L22 |
| ECs4181 | 4185263 | 4185538 | 30S ribosomal subunit protein S19 |
| ECs4182 | 4185558 | 4186376 | 50S ribosomal subunit protein L2 |
| ECs4183 | 4186397 | 4186696 | 50S ribosomal subunit protein L23 |
| ECs4184 | 4186696 | 4187298 | 50S ribosomal subunit protein L4, regulates expression of S10 operon |
| ECs4185 | 4187312 | 4187938 | 50S ribosomal subunit protein L3 |
| ECs4191 | 4191309 | 4193420 | GTP-binding protein chain elongation factor EF-G |
| ECs4193 | 4194087 | 4194458 | 30S ribosomal subunit protein S12 |
| ECs4194 | 4194587 | 4194871 | hypothetical protein |
| ECs4195 | 4194882 | 4195238 | hypothetical protein |
| ECs4198 | 4196516 | 4197325 | FKBP-type peptidyl-prolyl cis-trans isomerase |
| ECs4203 | 4201195 | 4203105 | putative ATP-binding component of a transport system |
| ECs4205 | 4204124 | 4204339 | hypothetical protein |
| ECs4206 | 4204396 | 4205262 | probable phosphoribulokinase |
| ECs4207 | 4205323 | 4205724 | hypothetical protein |
| ECs4208 | 4206026 | 4206655 | cyclic AMP receptor protein |
| ECs4211 | 4210175 | 4210735 | p-aminobenzoate synthetase component II |
| ECs4214 | 4211634 | 4212203 | peptidyl-prolyl cis-trans isomerase A |
| ECs4215 | 4212474 | 4213652 | putative transport |
| ECs4216 | 4213917 | 4216457 | nitrite reductase (NAD(P)H) subunit |
| ECs4217 | 4216457 | 4216780 | nitrite reductase (NAD(P)H) subunit |
| ECs4218 | 4216909 | 4217712 | nitrite reductase activity |
| ECs4219 | 4217734 | 4219104 | uroporphyrinogen III methylase |
| ECs4220 | 4219362 | 4219526 | hypothetical protein |
| ECs4226 | 4224833 | 4225834 | tryptophan tRNA synthetase |
| ECs4228 | 4226581 | 4227255 | D-ribulose-5-phosphate 3-epimerase |
| ECs4229 | 4227276 | 4228109 | DNA adenine methylase |
| ECs4231 | 4229597 | 4230682 | 3-dehydroquinate synthase |
| ECs4233 | 4231664 | 4232899 | putative transport portein |
| ECs4239 | 4237786 | 4238343 | hypothetical protein |
| ECs4240 | 4238663 | 4240795 | putative dehydrogenase |
| ECs4242 | 4241542 | 4241940 | ribosome-associated heat shock protein Hsp15 |
| ECs4247 | 4248062 | 4248778 | response regulator OmpR |
| ECs4250 | 4252338 | 4252562 | ferrous iron transport protein A |
| ECs4254 | 4257055 | 4257735 | hypothetical protein |
| ECs4256 | 4257797 | 4258369 | hypothetical protein |
| ECs4259 | 4262191 | 4264581 | maltodextrin phosphorylase |
| ECs4267 | 4273573 | 4274400 | protein of glp regulon |
| ECs4274 | 4282403 | 4283833 | glycogen synthase |
| ECs4276 | 4285149 | 4287119 | glycogen operon protein GlgX |
| ECs4277 | 4287119 | 4289302 | 1,4-alpha-glucan branching enzyme |
| ECs4279 | 4290870 | 4291460 | hypothetical protein |
| ECs4285 | 4294978 | 4296315 | low-affinity gluconate transport permease protein |
| ECs4287 | 4296988 | 4297980 | regulator of gluconate operon |
| ECs4288 | 4298207 | 4298899 | hypothetical protein |
| ECs4297 | 4306844 | 4307686 | integral membrane protein of sn-glycerol 3-phosphate transport system |
| ECs4298 | 4307686 | 4308570 | integral membrane protein of sn-glycerol 3-phosphate transport system |
| ECs4299 | 4308671 | 4309984 | periplasmic binding protein of sn-glycerol 3-phosphate transport system |
| ECs4302 | 4311927 | 4312691 | ATP-binding component of high-affinity branched-chain amino acid transport system |
| ECs4304 | 4313965 | 4314888 | membrane component of high-affinity branched-chain amino acid transport system |
| ECs4310 | 4319176 | 4320027 | RNA polymerase sigma32 factor |
| ECs4312 | 4321326 | 4321991 | ATP-binding component of a membrane-associated complex involved in cell division |
| ECs4314 | 4323640 | 4324233 | hypothetical protein |
| ECs4316 | 4324501 | 4324857 | putative receptor |
| ECs4317 | 4324998 | 4325621 | putative enzyme |
| ECs4342 | 4347574 | 4348158 | putative phosphopantetheinyltransferase |
| ECs4345 | 4350787 | 4351617 | transport of nickel, membrane protein |
| ECs4365 | 4372127 | 4373623 | low-affinity phosphate transport |
| ECs4366 | 4373700 | 4374032 | hypothetical protein |
| ECs4367 | 4374423 | 4374854 | universal stress protein UspA |
| ECs4368 | 4375174 | 4376640 | putative transport protein |
| ECs4378 | 4387796 | 4388323 | hypothetical protein |
| ECs4390 | 4398546 | 4398875 | hypothetical protein |
| ECs4391 | 4399130 | 4399699 | hypothetical protein |
| ECs4393 | 4401367 | 4402521 | putative membrane protein |
| ECs4395 | 4406028 | 4406753 | putative ARAC-type regulatory protein |
| ECs4400 | 4413432 | 4414031 | putative regulator |
| ECs4408 | 4424302 | 4425585 | uptake of C4-dicarboxylic acids |
| ECs4415 | 4438146 | 4438331 | hypothetical protein |
| ECs4421 | 4445014 | 4445994 | putative ATP-binding component of dipeptide transport system |
| ECs4423 | 4446920 | 4447936 | dipeptide transport system permease protein 1 |
| ECs4424 | 4448090 | 4449694 | dipeptide transport protein |
| ECs4435 | 4461226 | 4461663 | hypothetical protein |
| ECs4437 | 4464121 | 4464777 | putative outer membrane protein |
| ECs4440 | 4467052 | 4467339 | hypothetical protein |
| ECs4441 | 4467623 | 4467832 | cold shock protein 7.4 |
| ECs4443 | 4470580 | 4471488 | glycine tRNA synthetase alpha subunit |
| ECs4448 | 4475616 | 4476935 | D-xylose isomerase |
| ECs4453 | 4482438 | 4483259 | putative ATP-binding protein |
| ECs4477 | 4516384 | 4516968 | repressor for mtl |
| ECs4486 | 4529477 | 4530493 | glycerol-3-phosphate dehydrogenase (NAD+) |
| ECs4488 | 4531026 | 4531274 | glutaredoxin 3 |
| ECs4490 | 4532092 | 4533633 | putative 2,3-bisphosphoglycerate-independent phosphoglycerate mutase |
| ECs4492 | 4534933 | 4535889 | hypothetical protein |
| ECs4494 | 4537156 | 4538178 | threonine dehydrogenase |
| ECs4497 | 4540820 | 4541749 | ADP-L-glycero-D-mannoheptose-6-epimerase |
| ECs4498 | 4541762 | 4542805 | ADP-heptose--lps heptosyltransferase II |
| ECs4509 | 4553684 | 4554160 | phosphopantetheine adenylyltransferase |
| ECs4510 | 4554205 | 4555011 | formamidopyrimidine DNA glycosylase |
| ECs4511 | 4555112 | 4555276 | 50S ribosomal subunit protein L33 |
| ECs4512 | 4555300 | 4555533 | 50S ribosomal subunit protein L28 |
| ECs4516 | 4558353 | 4558946 | putative transcriptional regulator |
| ECs4517 | 4558989 | 4559627 | orotate phosphoribosyltransferase |
| ECs4524 | 4565968 | 4566240 | RNA polymerase omega subunit |
| ECs4526 | 4568377 | 4569063 | putative RNA methylase |
| ECs4603 | 4632911 | 4634299 | regulator of uhpT |
| ECs4606 | 4637271 | 4637858 | hypothetical protein |
| ECs4615 | 4645592 | 4646086 | hypothetical protein |
| ECs4616 | 4646086 | 4646445 | hypothetical protein |
| ECs4626 | 4657456 | 4657881 | heat shock protein IbpA |
| ECs4632 | 4662376 | 4663185 | hypothetical protein |
| ECs4635 | 4666384 | 4667454 | DNA polymerase III beta-subunit |
| ECs4640 | 4671309 | 4672952 | GTP-binding protein in thiophene and furan oxidation |
| ECs4650 | 4683361 | 4683924 | putative membrane / transport protein |
| ECs4651 | 4683984 | 4685318 | putative phosphatase |
| ECs4661 | 4694304 | 4695074 | phosphate transport system permease protein PstA |
| ECs4663 | 4696150 | 4697106 | phosphate-binding periplasmic protein PstS |
| ECs4673 | 4709000 | 4709416 | membrane-bound ATP synthase beta-subunit AtpD |
| ECs4676 | 4711763 | 4713301 | membrane-bound ATP synthase delta-subunit AtpH |
| ECs4677 | 4713317 | 4713847 | membrane-bound ATP synthase subunit b AtpF |
| ECs4678 | 4713865 | 4714332 | membrane-bound ATP synthase subunit c AtpE |
| ECs4679 | 4714397 | 4714633 | membrane-bound ATP synthase subunit a AtpB |
| ECs4680 | 4714683 | 4715495 | membrane-bound ATP synthase AtpI |
| ECs4683 | 4717191 | 4719077 | MioC protein |
| ECs4684 | 4719459 | 4719899 | transcriptional regulatory protein AsnC |
| ECs4686 | 4720599 | 4721588 | hypothetical protein |
| ECs4687 | 4721599 | 4723047 | putative 2-component regulator |
| ECs4689 | 4724760 | 4726625 | membrane-associated component of D-ribose high-affinity transport system |
| ECs4691 | 4727222 | 4728724 | D-ribose high-affinity transport system permease protein |
| ECs4697 | 4734082 | 4734771 | regulator of pssA |
| ECs4698 | 4740575 | 4741411 | hypothetical protein |
| ECs4699 | 4741530 | 4741865 | putative 2-component regulator |
| ECs4704 | 4745930 | 4746856 | dihydroxyacid dehydratase |
| ECs4709 | 4752940 | 4753218 | hypothetical protein |
| ECs4713 | 4757774 | 4759036 | thioredoxin 1 |
| ECs4721 | 4765928 | 4766992 | glucose-1-phosphate thymidylyltransferase |
| ECs4722 | 4767014 | 4767892 | hypothetical protein |
| ECs4724 | 4768552 | 4769679 | putative cytochrome |
| ECs4727 | 4772007 | 4773356 | probable UDP-N-acetyl-D-mannosaminuronic acid transferase |
| ECs4728 | 4773362 | 4774099 | putative amino acid/amine transport protein |
| ECs4729 | 4774293 | 4775675 | putative arylsulfatase regulator |
| ECs4732 | 4780329 | 4781522 | uroporphyrinogen III methylase |
| ECs4734 | 4782749 | 4783486 | porphobilinogen deaminase |
| ECs4738 | 4787400 | 4787717 | diaminopimelate epimerase |
| ECs4746 | 4795967 | 4796914 | hypothetical protein |
| ECs4755 | 4805480 | 4806499 | hypothetical protein |
| ECs4762 | 4813642 | 4815066 | ubiquinone/menaquinone biosynthesis methyltransferase |
| ECs4763 | 4815164 | 4815916 | hypothetical protein |
| ECs4765 | 4816535 | 4818172 | Sec-independent protein translocase |
| ECs4767 | 4818527 | 4819039 | Sec-independent protein translocase |
| ECs4770 | 4820645 | 4821130 | putative oxidoreductase |
| ECs4775 | 4827281 | 4828609 | hypothetical protein |
| ECs4777 | 4829265 | 4830713 | protoporphyrin oxidase |
| ECs4782 | 4838367 | 4839350 | protein disulfide isomerase I |
| ECs4783 | 4839370 | 4839993 | putative GTP-binding protein |
| ECs4792 | 4852427 | 4853833 | putative GTP-binding factor |
| ECs4793 | 4854206 | 4856026 | putative transcriptional regulator |
| ECs4809 | 4872717 | 4873586 | D-Tyr-tRNATyr deacylase |
| ECs4810 | 4873586 | 4874020 | putative acetyltransferase |
| ECs4817 | 4878938 | 4879864 | formate dehydrogenase cytochrome B556 (FDO) subunit |
| ECs4818 | 4879864 | 4880496 | formate dehydrogenase-O iron-sulfur subunit |
| ECs4819 | 4880496 | 4881395 | formate dehydrogenase-O major subunit |
| ECs4831 | 4895942 | 4897408 | Positive regulator for rhaBAD operon |
| ECs4840 | 4906429 | 4907328 | 6-phosphofructokinase I |
| ECs4842 | 4908793 | 4909779 | CDP-diacylglycerol phosphotidylhydrolase |
| ECs4844 | 4910702 | 4911466 | hypothetical protein |
| ECs4846 | 4912274 | 4912711 | hypothetical protein |
| ECs4847 | 4912926 | 4913222 | putative regulator |
| ECs4850 | 4914531 | 4915538 | glycerol kinase |
| ECs4852 | 4917302 | 4918144 | hypothetical protein |
| ECs4859 | 4923252 | 4923779 | essential cell division protein |
| ECs4870 | 4939498 | 4940385 | hydroperoxidase HPI(I) |
| ECs4873 | 4943926 | 4944540 | glycerol dehydrogenase |
| ECs4879 | 4950506 | 4950823 | formate acetyltransferase 2 |
| ECs4882 | 4954020 | 4954358 | putative ARAC-type regulatory protein |
| ECs4885 | 4957333 | 4959981 | acetylornithine deacetylase |
| ECs4887 | 4961638 | 4962639 | acetylglutamate kinase |
| ECs4890 | 4965127 | 4966041 | soluble pyridine nucleotide transhydrogenase |
| ECs4891 | 4966030 | 4967427 | putative amino acid amidohydrolase |
| ECs4895 | 4971036 | 4971392 | tRNA (uracil-5-)-methyltransferase |
| ECs4904 | 4986375 | 4986755 | component in transcription antitermination |
| ECs4905 | 4986760 | 4987302 | 50S ribosomal subunit protein L11 |
| ECs4906 | 4987464 | 4987889 | 50S ribosomal subunit protein L1 |
| ECs4907 | 4987896 | 4988597 | 50S ribosomal subunit protein L10 |
| ECs4908 | 4988892 | 4989386 | 50S ribosomal subunit protein L7/L12 |
| ECs4909 | 4989456 | 4989818 | RNA polymerase beta subunit |
| ECs4911 | 4994246 | 4998466 | heat shock protein htrC |
| ECs4914 | 5000693 | 5001460 | thiamin biosynthesis protein ThiG |
| ECs4918 | 5005160 | 5005633 | putative transcriptional regulator |
| ECs4919 | 5005728 | 5006498 | hypothetical protein |
| ECs4921 | 5007615 | 5008283 | endonuclease V |
| ECs4922 | 5008329 | 5008916 | hypothetical protein |
| ECs4923 | 5009106 | 5009375 | DNA-binding protein HU-alpha |
| ECs4931 | 5022680 | 5023606 | homoserine transsuccinylase |
| ECs4938 | 5036132 | 5037760 | putative alpha helix protein |
| ECs4939 | 5037857 | 5038543 | peptidase E |
| ECs5007 | 5085147 | 5086493 | lysine sensitive aspartokinase III |
| ECs5012 | 5090908 | 5093001 | hypothetical protein |
| ECs5013 | 5093599 | 5094006 | hypothetical protein |
| ECs5022 | 5105688 | 5106182 | chorismate lyase |
| ECs5023 | 5106198 | 5107067 | 4-hydroxybenzoate-octaprenyltransferase |
| ECs5025 | 5109819 | 5110184 | diacylglycerol kinase |
| ECs5026 | 5110297 | 5110902 | regulator for SOS regulon |
| ECs5037 | 5122560 | 5123270 | diadenosine tetraphosphatase |
| ECs5039 | 5123804 | 5124157 | hypothetical protein |
| ECs5040 | 5124198 | 5127017 | excision nuclease subunit A |
| ECs5041 | 5127272 | 5127805 | ssDNA-binding protein |
| ECs5045 | 5130615 | 5131076 | redox-sensing activator of soxS |
| ECs5046 | 5131625 | 5132971 | hypothetical protein |
| ECs5047 | 5133125 | 5134771 | hypothetical protein |
| ECs5051 | 5138565 | 5140520 | acetyl-CoA synthetase |
| ECs5054 | 5142957 | 5143625 | formate-dependent nitrite reductase NrfC |
| ECs5083 | 5175521 | 5176102 | phosphonate metabolism |
| ECs5097 | 5190146 | 5191480 | putative amino acid/amine transport protein |
| ECs5098 | 5191620 | 5192378 | putative ARAC-type regulatory protein |
| ECs5100 | 5195172 | 5196077 | regulator of melibiose operon |
| ECs5101 | 5196360 | 5197712 | alpha-galactosidase |
| ECs5104 | 5200133 | 5201776 | fumarase B |
| ECs5106 | 5203768 | 5204484 | two-component transcriptional regulator DcuR |
| ECs5107 | 5204484 | 5206112 | two-component sensor protein DcuS |
| ECs5108 | 5206293 | 5206520 | hypothetical protein |
| ECs5109 | 5206535 | 5206804 | hypothetical protein |
| ECs5117 | 5217799 | 5219493 | thiol:disulfide interchange protein |
| ECs5118 | 5219472 | 5219807 | divalent cation tolerance protein CutA |
| ECs5123 | 5225139 | 5225429 | chaperonin GroES |
| ECs5127 | 5228913 | 5229938 | hypothetical protein |
| ECs5128 | 5229980 | 5230543 | elongation factor P |
| ECs5134 | 5234066 | 5234797 | iron-sulfur protein subunit of fumarate reductase FrdB |
| ECs5136 | 5236923 | 5237897 | putative lysyl-tRNA synthetase |
| ECs5139 | 5243120 | 5244085 | phosphatidylserine decarboxylase |
| ECs5144 | 5249310 | 5249768 | hypothetical protein |
| ECs5147 | 5252977 | 5253924 | delta(2)-isopentenylpyrophosphate tRNA-adenosine transferase |
| ECs5148 | 5254013 | 5254318 | host factor I for bacteriophage Q beta replication |
| ECs5150 | 5255764 | 5257020 | protease specific for phage lambda cII repressor |
| ECs5151 | 5257026 | 5258027 | protease specific for phage lambda cII repressor |
| ECs5152 | 5258112 | 5258306 | hypothetical protein |
| ECs5153 | 5258413 | 5259708 | adenylosuccinate synthetase |
| ECs5154 | 5259916 | 5260338 | hypothetical protein |
| ECs5157 | 5263860 | 5264258 | hypothetical protein |
| ECs5158 | 5264280 | 5264975 | putative alpha helical protein |
| ECs5166 | 5270679 | 5271425 | hypothetical protein |
| ECs5167 | 5271428 | 5272180 | putative DEOR-type transcriptional regulator |
| ECs5172 | 5275913 | 5276560 | probable hexulose-6-phosphate synthase |
| ECs5173 | 5276573 | 5277424 | putative hexulose-6-phosphate isomerase |
| ECs5175 | 5278245 | 5278517 | hypothetical protein |
| ECs5178 | 5279565 | 5279789 | 30S ribosomal subunit protein S18 |
| ECs5179 | 5279834 | 5280280 | 50S ribosomal subunit protein L9 |
| ECs5185 | 5284409 | 5285026 | FKBP-type 22KD peptidyl-prolyl cis-trans isomerase |
| ECs5192 | 5292218 | 5292955 | ammonium transport system structural protein |
| ECs5196 | 5295355 | 5296695 | putative transport protein |
| ECs5198 | 5297862 | 5299592 | hypothetical protein |
| ECs5200 | 5303374 | 5303712 | hypothetical protein |
| ECs5204 | 5304605 | 5305132 | inorganic pyrophosphatase |
| ECs5205 | 5305442 | 5306395 | putative LACI-type transcriptional regulator |
| ECs5210 | 5311265 | 5312635 | putative ligase |
| ECs5211 | 5312797 | 5313345 | putative alpha helix protein |
| ECs5216 | 5318595 | 5320247 | trehalase 6-P hydrolase |
| ECs5218 | 5321840 | 5322784 | repressor of treA,B,C |
| ECs5221 | 5326527 | 5326985 | aspartate carbamoyltransferase regulatory subunit |
| ECs5222 | 5327001 | 5327933 | aspartate carbamoyltransferase catalytic subunit |
| ECs5235 | 5336382 | 5339234 | valine tRNA synthetase |
| ECs5236 | 5339237 | 5339677 | DNA polymerase III chi subunit |
| ECs5237 | 5340034 | 5341542 | aminopeptidase A/I |
| ECs5281 | 5406458 | 5407639 | mannonate hydrolase |
| ECs5287 | 5414364 | 5414822 | hypothetical protein |
| ECs5321 | 5458599 | 5459333 | chromosome replication protein DnaC |
| ECs5327 | 5463238 | 5464023 | ferric iron reductase protein |
| ECs5330 | 5465849 | 5466259 | DNA polymerase III psi subunit |
| ECs5331 | 5466231 | 5466674 | acyltransferase for 30S ribosomal subunit protein S18 |
| ECs5343 | 5478724 | 5479440 | purine-nucleoside phosphorylase |
| ECs5346 | 5481693 | 5482658 | 3-phosphoserine phosphatase |
| ECs5354 | 5491242 | 5492108 | right origin-binding protein |
| ECs5355 | 5492319 | 5492789 | hypothetical protein |
| ECs5356 | 5492805 | 5493491 | catabolic regulation response regulator |
| ECs5357 | 5493494 | 5494915 | catabolite repression sensor kinase for PhoB |
| ECs5359 | 5496391 | 5497104 | Negative response regulator of genes in aerobic pathways ArcA |
